# Supplementary material for: Antenatal care processes in rural Southern Nepal: gaps in and quality of service provision—a cohort study
Source: BMJ Open. 2021 Dec 17;11(12):e056392. doi: 10.1136/bmjopen-2021-056392 (PMC8685974; doi:10.1136/bmjopen-2021-056392)
Supplement: Author's manuscript [file bmjopen-2021-056392.draft_revisions.pdf]

BMJ Open is committed to open peer review. As part of this commitment we make the peer review history of every article we publish publicly available.

When an article is published we post the peer reviewers' comments and the authors' responses online. We also post the versions of the paper that were used during peer review. These are the versions that the peer review comments apply to.

The versions of the paper that follow are the versions that were submitted during the peer review process. They are not the versions of record or the final published versions. They should not be cited or distributed as the published version of this manuscript.

BMJ Open is an open access journal and the full, final, typeset and author-corrected version of record of the manuscript is available on our site with no access controls, subscription charges or pay-per-view fees (http://bmjopen.bmj.com).

If you have any questions on BMJ Open's open peer review process please email info.bmjopen@bmj.com

BMJ Open

Antenatal care processes in rural Southern Nepal: gaps in and quality of service provision

Journal:	*BMJ Open*
Manuscript ID	bmjopen-2021-056392
Article Type:	Original research
Date Submitted by the Author:	18-Aug-2021
Complete List of Authors:	Bryce, Emily; Johns Hopkins University Bloomberg School of Public Health,<br>Katz, Joanne; Johns Hopkins University Bloomberg School of Public Health, International Health<br>Plama, Tsering; Johns Hopkins University Bloomberg School of Public Health, Department of International Health<br>Khatry, Subarna; Nepal Nutr Intervent Project Sarlahi<br>LeClerq, Steven; Johns Hopkins University Bloomberg School of Public Health, International Health; Nepal Nutr Intervention Project, Sarlahi<br>Munos, Melinda; Johns Hopkins Bloomberg School of Public Health
Keywords:	PUBLIC HEALTH, International health services < HEALTH SERVICES ADMINISTRATION & MANAGEMENT, Quality in health care < HEALTH SERVICES ADMINISTRATION & MANAGEMENT

SCHOLARONE™
Manuscripts

I, the Submitting Author has the right to grant and does grant on behalf of all authors of the Work (as defined in the below author licence), an exclusive licence and/or a non-exclusive licence for contributions from authors who are: i) UK Crown employees; ii) where BMJ has agreed a CC-BY licence shall apply, and/or iii) in accordance with the terms applicable for US Federal Government officers or employees acting as part of their official duties; on a worldwide, perpetual, irrevocable, royalty-free basis to BMJ Publishing Group Ltd ("BMJ") its licensees and where the relevant Journal is co-owned by BMJ to the co-owners of the Journal, to publish the Work in this journal and any other BMJ products and to exploit all rights, as set out in our licence.

The Submitting Author accepts and understands that any supply made under these terms is made by BMJ to the Submitting Author unless you are acting as an employee on behalf of your employer or a postgraduate student of an affiliated institution which is paying any applicable article publishing charge ("APC") for Open Access articles. Where the Submitting Author wishes to make the Work available on an Open Access basis (and intends to pay the relevant APC), the terms of reuse of such Open Access shall be governed by a Creative Commons licence – details of these licences and which Creative Commons licence will apply to this Work are set out in our licence referred to above.

Other than as permitted in any relevant BMJ Author's Self Archiving Policies, I confirm this Work has not been accepted for publication elsewhere, is not being considered for publication elsewhere and does not duplicate material already published. I confirm all authors consent to publication of this Work and authorise the granting of this licence.

For peer review only

Title: Antenatal care processes in rural Southern Nepal: gaps in and quality of service provision

Running Title: Quality of antenatal care in Southern Nepal

Authors: Emily Bryce, PhD[1], Joanne Katz, ScD[1] , Tsering Pema Lama, PhD[1,2], Subarna K. Khatry, MBBS, FRCS[2], Steven LeClerq, MPH[2] , Melinda Munos, PhD[1]

1-Johns Hopkins Bloomberg School of Public Health, Baltimore, Maryland USA 2- Nepal Nutrition Intervention Project, Sarlahi Nepal (NNIPS).

Corresponding author: Emily Bryce. 615 N Wolfe St, St Baltimore, MD 21205. (607) 592-5479. Email: ebryce2@jhu.edu

For peer review only

Word count: 4,170 (excluding headers and captions)
Number of references: 36
Number of Tables: 6
Number of Figures: 5

Conflict of interest: EB, MM, TL, SK, SLC and JK have no conflicts of interest.

Contribution statement: MM and JK designed the study. TL, SKK, SL, JK and EB contributed to the implementation of the study. EB conducted analyses and wrote the first draft of manuscript. MM and JK submitted edits to the manuscript. MM, JK, TL, SKK, EB and SL all have read and approve of the final version of the manuscript.

Funding: This research was funded by the Bill and Melinda Gates Foundation (grant number OPP1172551). The funders had no role in the design or implementation of the study, nor the analysis or interpretation of the data. JK and MM were the funding recipients

Data sharing: Data is available by emailing Joanne Katz, ScD or Emily Bryce, PhD

For peer review only - http://bmjopen.bmj.com/site/about/guidelines.xhtml

Abstract

Objective: This study aimed to compare a standard quality of care definition to one that reflected focused antenatal care (FANC) guidelines and examine associations with receipt of good quality of care.

Methods: In a longitudinal cohort study, we observed all the antenatal care visits 434 women attended at five government health posts in Southern Nepal. We examined differences in frequency of provision and service provision by gestational age at visit. We developed two quality of care definitions: 1) provision of seven services at least once during pregnancy and 2) provision of services to reflect the FANC guidelines by incorporating a frequency of care dimension for certain services. Multivariate log-binomial and Poisson with robust variance models were used to examine associations with receipt of good quality of care.

Findings: There was variation in service provision both in terms of frequency of provision and by gestational age at the visit. There were 213 women (49.1%) that received good quality care by the first definition, but when the frequency of service provision was included for the second definition the percentage dropped to 6.2%. There were significant differences in provision of quality care by health post. The number of visits, maternal age and care during the first trimester was associated with greater likelihood of good quality antenatal care.

Conclusion: This analysis demonstrated that measuring quality of care by receipt of services at least once during pregnancy may overestimate the true coverage of quality of antenatal care. Future efforts should improve feasibility of including frequency of care in quality of care definitions.

Strengths and limitations of this study

- Services received during antenatal care was established using direct observation rather than maternal report, thus eliminating the possibility the risk of recall bias
- Content of care was observed across the entire pregnancy, rather than cross sectionally at a single visit
- The study observers were required to meet inter and intra-observer reliability standards before study began
- Potential for observer effect, whereby providers altered their care because of the presence of the study observers

For peer review only - http://bmjopen.bmj.com/site/about/guidelines.xhtml

For peer review only

- Smaller sample size because of the resources required for the longitudinal nature of the study

For peer review only

Background

Despite the progress made in the previous two decades, in 2017 approximately 810 women died every day from preventable pregnancy and childbirth related causes.(1) The vast majority of these deaths occurred in low and lower-middle income countries. However, there are existing interventions that reduce maternal and neonatal mortality and morbidity that can be delivered during pregnancy.(2-5)

For peer review only - http://bmjopen.bmj.com/site/about/guidelines.xhtml

Along the reproductive health continuum, antenatal care (ANC) is defined as care provided during pregnancy by a skilled health provider, often at a first level facility.(3) ANC provides the opportunity to identify pregnancy risk factors through screening processes, prevent and manage diseases (pre-existing or pregnancy-related), and to provide health education and promotion.(3) Furthermore, good quality ANC has been associated with higher rates of facility delivery, which improves birth outcomes for mother and infant.(6, 7)

Guidelines for antenatal care have evolved over time, moving from a European model involving an average of twelve visits to the World Health Organization (WHO) four-visit, focused antenatal care model (FANC) in 2002 to the most current 2016 WHO guidelines requiring a minimum of eight visits during pregnancy.(3, 8) However, for many countries the transition to the eight-visit model has not yet been made. For example, Nepal's national protocol still follows the FANC model, which recommends four visits by four, six, eight and nine months gestational age.(9, 10) The country's safe motherhood program, *"Aama Suraksha"*, provides financial rewards to women who attended ANC during those four specified months and who deliver in a health facility.(9) Beyond the scheduling recommendations there are suggested components of ANC in Nepal, but there are no current, published guidelines; the last maternity guidelines were published in 1996.(11)

The coverage indicator ANC4+, or the proportion of pregnant women who attend four or more antenatal care visits, served as the measure of adequate antenatal care for the fifth Millennium Development Goal (MDG) and for countries to monitor their maternal care programs.(12) However, this indicator does not capture the content of the care provided, but rather contact with the health system. This is particularly problematic for ANC, as there are several interventions and health messages required for complete ANC.(3, 13, 14) Over the years, the measurement of ANC quality has shifted from contact-focused to content-focused.(12)

There is no single definition for "quality of care" for antenatal care, which complicates its measurement.(15) The WHO defines quality of care as healthcare that is "safe, effective, timely, efficient, equitable, and people-centered".(14) Accompanying this definition is a framework (Figure 1), which combines the Donabedian systems approach of evaluation (structure, process, outcome) and the two components of process quality (provision and experience of care) that are central components to other authors' definitions of quality of care.(14, 16-19)

In Nepal, 84% of women received any ANC from a skilled provider and 69% of women had at least four ANC visits, though the proportion is lower in rural areas.(20) 58.6% of women had a visit during the four months (4th, 6th, 8th and 9th) per national protocol. Although coverage of ANC has increased, as of 2016 the maternal mortality ratio was equal to 259 deaths per 100,000 live births, well above the SDG target of 70 deaths per 100,000 live births.(20) This indicates a gap in quality for provision of maternal care, including antenatal care.

For peer review only - http://bmjopen.bmj.com/site/about/guidelines.xhtml

A few other studies have examined the quality of antenatal care in Nepal, though some of the analyses use Demographic Health Survey data that is cross-sectional and subject to recall bias.(5, 21-23) To our knowledge, this is the first study in Nepal to use data generated by a direct observation during a longitudinal cohort study to examine antenatal care. Furthermore, the longitudinal nature of this study allows us to assess the frequency of service provision, as there are certain services (i.e. blood pressure and weight measurement) that should occur at every antenatal care visit. This study aims to describe the provision of antenatal care in our study population and to investigate factors associated with high quality ANC.

Methods

Data Sources

The data for this analysis were collected through the Coverage Validation Study, which was conducted within a part of the study area of the Nepal Nutrition Intervention Project Sarlahi (NNIPS) located in the Sarlahi District of Nepal. This study is one in a series of validation studies focused on measurement of nutrition-related indicators during pregnancy. The parent study aimed to enroll 450 women to end up with 300 women with complete antenatal care observation and completed follow-up interview for adequate power for the validation analyses.

Pregnant, married women, ages 15 and older who were presenting for their first antenatal care visit at one of five government health posts selected for this study were eligible to participate. If a woman presented after already attending an ANC visit or ultrasound appointment, she was deemed ineligible because the study would not be able to observe all ANC visits.

A demographic questionnaire was administered at the enrollment visit. The direct observation of the first and all subsequent ANC visits at the study health posts were conducted by trained study staff, using a checklist of 28 items. The socioeconomic and client satisfaction data were collected during a postpartum interview.

Process quality of care assessment

The aim of this analysis was to evaluate the quality of the processes of care provided during antenatal care. The process of care includes two components, provision of care and experience of care (Figure 1).

Given that Nepal's national protocol is based on four antenatal visits, the FANC model served as the standard for the provision of care. In addition to the FANC model, Nepal's Ministry of Health (MoH) annual report, a country-led quality analysis of the 2016 DHS and an expert survey were reviewed to determine final service inclusion (Table 1).(22, 24-26) The MoH report and 2016 DHS were referenced to include the

For peer review only - http://bmjopen.bmj.com/site/about/guidelines.xhtml

country's priorities and for comparison to other quality of care analyses. Additionally, the MoH annual report is the most recent documentation available with recommended antenatal care services in Nepal.

The provision of care domain was measured by receipt of the following: blood pressure measurement, weight measurement, IFA supplementation, blood test prescription, urine test prescription, deworming medication and counseling messages related to nutrition, breastfeeding, facility delivery and postpartum care. The prescription of blood and urine tests, rather than the performance of the test, was recorded because only one of the five health posts had a laboratory on-site for use. The parent study focused on nutrition-specific interventions, therefore we did not capture "counseling on complications and/or warning signs", which is commonly included in antenatal quality of care definitions (23, 27-29). Similarly, how the tetanus toxoid vaccine is distributed in this setting did not allow for its capture during observations and its inclusion in the quality of care metric.

We developed two quality of care definitions (Table 2). The process quality for the total study population is defined to align with other analyses of DHS or similarly collected cross-sectional data; at any point during a woman's pregnancy, she received any IFA (non-zero number of tablets), counseling in two of four areas (hypothesized to be dependent on gestational age), and the other five provision of care services at least once (QOC1). For the women who attended four or more ANC visits, quality was measured as at least 120 IFA tablets received, weight and BP measurements at least four times (as these services should be received at each ANC visit), counseling in all four areas and the remaining services at least once (QOC4+). Experience of care was measured the same in both groups.

Covariates

There were six maternal characteristics that were included to examine their association with quality of care: maternal age, maternal education, prior live births, trimester at enrollment household socioeconomic status and health post attendance. Maternal education was dichotomized in "zero years of education" and "any years of education" and prior live births into "no prior live births" and "one or more prior live birth". The trimester at enrollment was calculated by subtracting the date of reported last menstrual period (LMP) from the date of enrollment. Gestational age at each visit was calculated by subtracting the date of LMP from the visit date. These were then categorized into four groups (< 4 months, 4-6 months, 7-8 months and 9+ months) to reflect the FANC model groups. The household socioeconomic status was constructed through the summation of 11 variables (including number of rooms, fuel and water sources, latrine type and ownership variables) and dividing the total sum by the total number of non-missing responses. This proportion was then separated into quartiles. Nine of the 434 women (2%) attended more than one health post during observations; the remaining 98% of participants attended the same health post for all observations.

For peer review only - http://bmjopen.bmj.com/site/about/guidelines.xhtml

For those nine women, the health post they attended more frequently was considered their "primary health post".

Statistical analysis

Receipt of services was examined by gestational age using chi-square tests. Descriptive statistics were estimated to compare quality of care provided at each of the five health posts and between women of different characteristics using proportions for categorical variables and means for continuous variables.

Bivariate and multivariate log-binomial regression models were used to estimate risk ratios and the corresponding 95% confidence intervals for the associations between receipt of 1) QOC1 and 2) QOC4+. Relative risk estimates were calculated instead of odds ratios because the outcome was not rare (>10% of women received good quality of care) and therefore an odds ratio would have overestimated the relationships being examined If the log-binomial regression model did not converge, a poisson model with robust error variance was used. All analyses were conducted using Stata version 14.2 (Stat Corp, College Station, Texas).

Patient and Public Involvement

Study participants were not involved in this design, recruitment, conduct or dissemination of this research. The 28-item checklist was reviewed by a local community advisory board in Nepal before the start of the study, but the public had no other part in the development or implementation of this study. There are no plans to disseminate results to the participants or community, aside from the local study staff who reside in the community.

Ethical approval statement

The Institutional Review Board of the Johns Hopkins Bloomberg School of Public Health (Baltimore, Maryland) and the National Health Research Council (Kathmandu, Nepal) approved this study. Informed consent was obtained from providers and participants at the beginning of the study and participants were re-consented at the postpartum interview.

Results

A total of 441 women were enrolled in the study and seven women (1.6% of the participants) were lost to follow-up for the six-month postpartum interview, resulting in a final sample of 434 women. The women were between 16 and 41 years old at enrollment, with an average age of 22.5 years (Table 3). Sixty percent had 4 or more ANC visits and 31% of women attended a visit during each of the four recommended

For peer review only

months outlined by the FANC model. Sixty percent of the women reported no years of education and 32% had no prior live births.

Frequency of an intervention or counseling message being observed, categorized as never, once or more than once and the reported experience of care is shown in Table 4. The majority of IFA and medication-related interventions were received once or more than once. Four women (0.9%) were observed receiving deworming medication twice, which is outside recommendations. The majority of women had blood pressure and weight measured more than once (78.1% and 81.3%, respectively). Over 70% of women did not receive any counseling regarding breastfeeding and 67.5% never received advice on postpartum related subjects (e.g., importance of postpartum visits, when to come for the visit). Eighty-seven percent of women reported being able to discuss problems and concerns with their provider and 97.8% reported somewhat or very respectful care.

There were no differences in the measurement of or advice concerning blood pressure or weight by gestational age at the visit (Table 5). The proportion of women receiving the service in each of the four categories was significantly different for all other services. A greater proportion of women beyond four months gestational age received IFA tablets and were given or told to buy calcium tablets than less than four months. Conversely, a higher proportion of women received deworming medication and prescriptions for blood and urine tests at visits that occurred at less than four months and between four to six months. Nutrition-related advice and counseling on ceasing smoking and drinking was more frequently provided at visits earlier in pregnancy. Advice on matters more related to delivery (breastfeeding, delivery in a facility) and postpartum subjects were given to a higher proportion of women at nine months gestational age or greater.

Quality of care

There were 213 women (49.1%) of the total 434 that were observed receiving good quality of antenatal care by the first definition (QOC1). The proportion significantly decreased to 10.3% (27 of 262 women) when the second definition was applied among women with four or more visits (QOC4+); 6.2% of the entire cohort. There was significant variation between health posts and the proportion of women receiving good quality of care (Figures 2a & 2b). The second health post, at which the greatest number of women were enrolled, did not have any women with four or more visits receive good quality of care. The proportion of women that received each element of the quality of care definitions by health post are presented in Figures 3a and 3b to illustrate gaps in provision. Across the majority of the health posts, the element with the lowest prevalence was the receipt of the appropriate number of counseling messages. While any IFA supplementation for QOC1 is not a limiting factor, the proportion of women who received the requisite 120+ tablets for QOC4+ is much lower across all health posts.

For peer review only - http://bmjopen.bmj.com/site/about/guidelines.xhtml

Table 6 presents the bivariate and multivariate regression results. The greater the number of ANC visits a woman attended was associated with improved quality of care by both definitions (QOC1 aRR= 1.18 [1.13-2.13, p<0.01]; QOC4+ aRR= 1.46 [1.11-2.80, p<0.01]). Additionally, women who were 20 years old or younger had 27% greater likelihood of receiving QOC1 than older women. Women who attended their first ANC visit during the first trimester were 22% more likely to have received QOC1. The direction and magnitude of these two covariate's associations with QOC4+ were similar to QOC1, but they were not statistically significant.

Discussion

This paper examined the process and quality of antenatal care in Southern Nepal using data from a longitudinal study of direct antenatal care observations. The analyses reveal there are gaps in provision of care and differences in services received by gestational age at visit. Nearly half of women received good quality of antenatal care defined by receipt of the services at least once during pregnancy and a good experience of care. However, when the QOC definition was expanded to reflect the FANC model recommendations, the percent drops to 6.2% of women receiving good quality of care. While over 60% of the women attended at least four ANC visits, only 31% attended a visit during each of the four recommended gestational age periods as outlined by the FANC model. This illustrates that aside from not capturing the content of care, the ANC4+ indicator may a poor measure of the recommended contacts for measuring antenatal care coverage.

The results show a gap in care for counseling during ANC, specifically messaging pertaining to breastfeeding and postpartum visits. Low rates of counseling in these areas have been documented in other studies of quality of antenatal care as well.(27, 29, 30) The high rate of counseling about nutrition in our study is similar to another in Nepal using DHS data.(23) The lower rates of counseling on facility delivery, breastfeeding and postpartum visits in our setting could be because the receipt of financial incentives for the safe motherhood program is contingent on facility delivery and it is assumed these messages will be delivered at that time. However, rates of facility delivery in Nepal are still quite low, 56% in 2016,(20) therefore these aspects of counseling should also be covered during antenatal care. Additionally, a recent review also found major gaps in quality of counseling messaging in Nepal and four other South Asian countries.(31) We did not measure the quality of the counseling messaging themselves, only their delivery. However, Torlesse et al.'s findings may mean that the coverage measures in our population may overestimate the proportion of women who received quality counseling that led to information transfer from provider to client.

Aside from weight and blood pressure measurement, which should be completed at every visit, there were significant differences in the proportion of women receiving a service at a given gestational age. This corresponds with findings from studies in Pakistan and Bangladesh.(28, 32) The timing is a critical component of care because certain screening tests (i.e., blood test for syphilis) and IFA supplementation should be

For peer review only - http://bmjopen.bmj.com/site/about/guidelines.xhtml

received earlier in pregnancy.(3) Furthermore, this has implications for women who do not attend ANC during all recommended months and may miss components of care.

The definition of quality of antenatal care is commonly the receipt of certain services at any point during pregnancy. Our data shows that using this definition, 49.1% of the women in our population received good quality of antenatal care. This is higher than another study using 2011 Nepal DHS data,(23) and studies conducted India, Bangladesh and in sub-Saharan Africa.(27, 28, 33, 34) A possible explanation could be that as our data is more recent, health service delivery has improved since the previous studies. Additionally, the other studies primarily rely on maternal recall of services received during ANC, many of which have yet to be validated, which could lead to reporting errors. Finally, our coverage of QOC1 could be higher due to the observer effect (discussed in full in limitations), resulting in the providers at the study clinics providing more complete care than they would if not under observation.

To our knowledge, this is the first study in a low-and-middle-income setting to examine quality of care by including frequency of service provision as outlined by the FANC model. A study in Kenya asked women to report whether they had their weight and blood pressure measured “never, a few times, most of the time and all the time” and found the majority of women to have received the two services most or all of the time.(29) This is comparable to our findings, but this study did not create a final QOC metric to which we can compare our QOC4+ results.

Our analysis showed that the proportion of women received good quality of care differed by health post, more so for QOC4+ than for QOC1. A multi-level model with a random intercept for health post and inclusion of health post level covariates would have been ideal for further elucidating reasons for these differences. However, with only five health posts, the number of clusters was too small to conduct these analyses. Exploring associations between provider characteristics and quality of care would be helpful to explain differences in provision, however the parent study did not collect this information.

The total number of ANC visits is the only characteristic that is significantly associated with both of the QOC definitions, which is consistent with another study.(33) The greater the number of visits a woman attended, the greater the opportunity to have received all necessary components. Younger women (<20 years) were more likely to have received better quality of care, which was not the case in other studies in Nepal and Kenya.(23, 29)

There were no differences in QOC by SES quartile in our study, whereas others have shown there to be a significant relationship between SES and QOC.(23, 33, 35) However, these studies primarily use data from population-based surveys (e.g., DHS) and have a more heterogeneous population in regard to SES. In our population, the mean score was 6.3 (SD=2.3) and was skewed towards lower scores. There was no difference in QOC1 by education, which has been demonstrated in some studies (29, 36) but not others.(23, 27, 33) As with socioeconomic status, our population was more homogenous in educational attainment; 60% of the population reported no years of

For peer review only - http://bmjopen.bmj.com/site/about/guidelines.xhtml

education and only one woman reported schooling beyond grade school, compared to 38.9% with no education and 7% with tertiary education in the 2011 Nepal DHS study.(23)

There were no statistically significant associations between covariates and QOC4+, which could be due to a lack of power (discussed further in limitations), as the magnitude of the coefficient for many is quite large. While we cannot draw conclusions on associations between the covariates and the QOC4+ outcome, the descriptive analyses demonstrate the gaps in care and difference in coverage between QOC4+ and QOC1. The health post with the greatest number of patients had no women with high quality QOC4+, driven primarily by a lack of counseling. This health post also had the smallest proportion of women who received 120+ tablets and deworming medication. The increased patient load could explain the low proportions of women receiving the commodity-based services; stock outs might have occurred, or the providers may have limited the number of IFA tablets given to a woman at any one visit to delay a stockout. The lack of counseling could be due to time limitations; the provider may feel that they cannot spend adequate time to convey all the health education messaging because of waiting patients. However, the satisfaction score for this health post was high and similar to the other posts.

A strength of this study is that the receipt of services was collected by direct observation, rather than maternal report. The use of direct observation removes the possibility for recall bias and social desirability bias from the participant, both of which can be present in the cross-sectional studies commonly used in antenatal quality of care studies. Furthermore, the longitudinal design of the parent study allowed for the observation of a woman's entire pregnancy and record of frequency of service provision. Another strength includes the use of trained observers who were required to meet inter and intra-observer reliability standards before the study began.

There are limitations to this analysis as well. While the data is not subject to recall or social desirability bias, the presence of the study observers during ANC could have elicited an observer effect, where the providers knew they were being observed and altered their care. To reduce this possibility, providers were told during the consent process that observers are not medically trained and will not know if the procedures they are observing are correct or not, nor will they report what they observe to the clinic supervisor. Additionally, the observers were present for over a year and one would hope that any observer effect would dissipate over the extended time period. The client satisfaction data was collected at the postpartum interview, which means it is subject to recall and social desirability bias. However, there has been evidence of "courtesy bias", where individuals are less likely to report critically at the facility, which is avoided in this case. Additionally, gestational age estimation was based on maternal report of last menstrual period, which is subject to recall bias as well. Eighty-one percent of the sample presented by the fourth month of pregnancy, so the hope is that with a shorter recall period the LMP data is still fairly accurate. Another limitation was that we only observed women who presented for antenatal care at government health facilities. Therefore, the generalizability may be limited to women who attend antenatal care or

For peer review only - http://bmjopen.bmj.com/site/about/guidelines.xhtml

those who remain within the system in which we observed (excluding private facilities, traditional healers, etc,). A final limitation is the smaller sample size compared other ANC QOC studies, which may have led to limited power in detecting associations between QOC and maternal and facility characteristics. However, because the longitudinal direct observation design is so time and resource intensive, it would be difficult to get a much larger sample size.

This analysis demonstrates that measuring quality of care by receipt of services at least once during pregnancy may be overestimating the true coverage of quality of antenatal care. Furthermore, as countries transition to the eight-visit 2016 WHO ANC recommendations, the degree of underestimation will only increase. Additionally, despite a common belief that commodity-based services such as IFA tablets are often the limiting factor for quality of care, in this study the largest gap in service provision was seen for adequate counseling messaging and the counseling adequacy varied significantly across the study health posts. Future programming and policy efforts should fortify provider training on effective counseling delivery and interpersonal skills to close the gap in counseling provision. This analysis supports the continued research and programmatic efforts in strengthening of routine health information systems and electronic health records to measure quality of care over a woman's entire pregnancy. This would allow the frequency of service provision to be included in quality of care assessment. Additionally, these systems and household surveys should be updated to capture additional indicators of counseling delivery. The inclusion of frequency of specific services provided in ANC in quality of care measurement may be the next step in closing the "quality-coverage" gap and achieving optimal maternal, newborn and child survival.

Conflict of interest: EB, MM, TPL, SK, SLC and JK have no conflicts of interest.

Contribution statement: MM and JK designed the study. TPL, SKK, SL, JK and EB contributed to the implementation of the study. EB conducted analyses and wrote the first draft of manuscript. MM and JK submitted edits to the manuscript. MM, JK, TPL, SKK, EB and SL all have read and approve of the final version of the manuscript.

Funding: This research was funded by the Bill and Melinda Gates Foundation (grant number OPP1172551). The funders had no role in the design or implementation of the study, nor the analysis or interpretation of the data. JK and MM were the funding recipients

Data sharing: Data is available by emailing Joanne Katz, ScD or Emily Bryce, PhD

Figure legends/caption:

For peer review only - http://bmjopen.bmj.com/site/about/guidelines.xhtml

Figure 1 caption: *Source: Tuncalp, Ö et al. Quality of care for pregnant women and newborns-the WHO vision(14)*

Figure 2a and 2b: N/A

Figure 3a legend:

	Deworming		Urine sample
	Blood sample		Weight
	Blood Pressure		2+ counseling
	Any IFA		Discuss problems
	Respectful care		

Figure 3b legend:

	Deworming		Urine sample
	Blood sample		Weight
	Blood Pressure		≥ 4 counseling
	≥ 120 IFA		Discuss problems
	Respectful care		

For peer review only

References

1. Trends in maternal mortality 2000 to 2017: estimates by WHO, UNICEF, UNFPA, World Bank Group and the United Nations Population Division. Geneva: World Health Organization; 2019.
2. Carroli G, Rooney C, Villar J. How effective is antenatal care in preventing maternal mortality and serious morbidity? An overview of the evidence. Paediatric and perinatal epidemiology. 2001;15 Suppl 1:1-42.
3. WHO. WHO recommendations on antenatal care for a positive pregnancy experience. Geneva, Switzerland; 2016.
4. Kuhnt J, Vollmer S. Antenatal care services and its implications for vital and health outcomes of children: evidence from 193 surveys in 69 low-income and middle-income countries. BMJ Open. 2017;7(11):e017122.
5. Neupane S, Nwaru BI. Impact of prenatal care utilization on infant care practices in Nepal: a national representative cross-sectional survey. European journal of pediatrics. 2014;173(1):99-109.
6. Guliani H, Sepehri A, Serieux J. What impact does contact with the prenatal care system have on women's use of facility delivery? Evidence from low-income countries. Soc Sci Med. 2012;74(12):1882-90.
7. Bloom SS, Lippeveld T, Wypij D. Does antenatal care make a difference to safe delivery? A study in urban Uttar Pradesh, India. Health Policy Plan. 1999;14(1):38-48.

8. Villar J, Ba'aqeel H, Piaggio G, et al. WHO antenatal care randomised trial for the evaluation of a new model of routine antenatal care. Lancet (London, England). 2001;357(9268):1551-64.
9. Pandey S. Women's knowledge about the conditional cash incentive program and its association with institutional delivery in Nepal. PloS one. 2018;13(6):e0199230.
10. WHO. WHO antenatal care randomized trial: manual for the implementation of the new model. Geneva; 2002.
11. Jahn A, Dar Iang M, Shah U, et al. Maternity care in rural Nepal: a health service analysis. Tropical medicine & international health : TM & IH. 2000;5(9):657-65.
12. Hodgins S, D'Agostino A. The quality-coverage gap in antenatal care: toward better measurement of effective coverage. Glob Health Sci Pract. 2014;2(2):173-81.
13. Bhutta ZA, Darmstadt GL, Hasan BS, et al. Community-based interventions for improving perinatal and neonatal health outcomes in developing countries: a review of the evidence. Pediatrics. 2005;115(2 Suppl):519-617.
14. Tuncalp, Were WM, MacLennan C, et al. Quality of care for pregnant women and newborns-the WHO vision. BJOG : an international journal of obstetrics and gynaecology. 2015;122(8):1045-9.
15. Raven JH, Tolhurst RJ, Tang S, et al. What is quality in maternal and neonatal health care? Midwifery. 2012;28(5):e676-83.
16. Donabedian A. Exploratings in quality assessment and monitoring definition of quality and approaches to its assessment. 1980.
17. Donabedian A. The quality of care. How can it be assessed? Jama. 1988;260(12):1743-8.
18. Hulton L, Matthews Z, Stones RW. A framework for the evaluation of quality of care in maternity services. 2000.
19. Bruce J. Fundamental elements of the quality of care: a simple framework. Stud Fam Plann. 1990;21(2):61-91.
20. Nepal MoH. Nepal Demographic and Health Survey 2016. Kathmandu, Nepal: Ministry of Health, Nepal; 2017.
21. Aryal K, Sharad SK, Mukti NK, Bihungum B, Shiv SL, Shambhu K, and Mona SM. Maternal Health Care in Nepal: Trends and Determinants. Rockville, Maryland, USA: ICF; 2019.
22. Lama TP, M. Chaulagain, A. Rai, and S. Gurung. Assessment of the Quality of Antenatal Care, Family Planning, and Sick Child Care Services in Nepal: Further Analysis of the 2015 Nepal Health Facility Survey. Rockville, Maryland, USA: ICF; 2019.
23. Joshi C, Torvaldsen S, Hodgson R, et al. Factors associated with the use and quality of antenatal care in Nepal: a population-based study using the demographic and health survey data. BMC pregnancy and childbirth. 2014;14:94.
24. Population MoHa. Annual Report, Department of Health Services 2074/2075 (2017/2018). Kathmandu, Nepal; 2019.
25. Population MoHa. Annual Report, Department of Health Services 2070/71 (2013/2014). Kathmandu, Nepal; 2015.
26. Sheffel A, Zeger S, Heidkamp R, et al. Development of summary indices of antenatal care service quality in Haiti, Malawi and Tanzania. BMJ Open. 2019;9(12):e032558.

For peer review only - http://bmjopen.bmj.com/site/about/guidelines.xhtml

27. Islam MM, Masud MS. Determinants of frequency and contents of antenatal care visits in Bangladesh: Assessing the extent of compliance with the WHO recommendations. PloS one. 2018;13(9):e0204752.
28. Siddique AB, Perkins J, Mazumder T, et al. Antenatal care in rural Bangladesh: Gaps in adequate coverage and content. PloS one. 2018;13(11):e0205149.
29. Afulani PA, Buback L, Essandoh F, et al. Quality of antenatal care and associated factors in a rural county in Kenya: an assessment of service provision and experience dimensions. BMC Health Serv Res. 2019;19(1):684.
30. Marchant T, Tilley-Gyado RD, Tessema T, et al. Adding content to contacts: measurement of high quality contacts for maternal and newborn health in Ethiopia, north east Nigeria, and Uttar Pradesh, India. PloS one. 2015;10(5):e0126840.
31. Andrews RM, McCarthy J, Carapetis JR, et al. Skin Disorders, Including Pyoderma, Scabies, and Tinea Infections. Pediatric Clinics of North America. 2009;56(6):1421-40.
32. Agha S, Tappis H. The timing of antenatal care initiation and the content of care in Sindh, Pakistan. BMC pregnancy and childbirth. 2016;16(1):190.
33. Olorunsaiye CZ, Brunner Huber LR, Laditka SB, et al. Individual and community socioeconomic factors related to the quality of antenatal care: a multilevel analysis of West and Central Africa. Women Health. 2021;61(1):15-26.
34. Singh L, Dubey R, Singh S, et al. Measuring quality of antenatal care: a secondary analysis of national survey data from India. BJOG : an international journal of obstetrics and gynaecology. 2019;126 Suppl 4:7-13.
35. Sharma J, Leslie HH, Kundu F, et al. Poor Quality for Poor Women? Inequities in the Quality of Antenatal and Delivery Care in Kenya. PloS one. 2017;12(1):e0171236.
36. Lee E, Madhavan S, Bauhoff S. Levels and variations in the quality of facility-based antenatal care in Kenya: evidence from the 2010 service provision assessment. Health Policy Plan. 2016;31(6):777-84.

For peer review only

Table 1

	FANC	Nepal MoH annual report	Nepal DHS analyses	Expert survey	What CVS-2 captured
Provision of care					
IFA	X	X	X	X	X
IPTp	X			(not for Nepal)	
TT vaccine	X	X	X	X	
Deworming		X	X	X	X
BP measured	X	X (2017 report)	X	X	X

For peer review only - http://bmjopen.bmj.com/site/about/guidelines.xhtml

Weight measured			X		X
Blood sample	X		X	X	X
Urine sample	X		X	X	X
Counseling	X	X	X	X	X
Nutrition	X	X	X	X	X
Breastfeeding	X			X	X
Facility delivery	X	X	X		X
Family planning			X		
PP care		X			X
Experience of care					
Ability to discuss problems			X	X	X
Respectful care			X	X	X
Satisfied with explanation of information			X	X	

Table 2. Quality of care definitions

	Definition 1: Entire study population (QOC1)	Definition 2: Women who had recommended 4+ visits (QOC4+)
Provision of care		
IFA	Any	120+ (30/visit)**
Deworming	Once	Once
BP measured	Once	Four times**
Weight measured	Once	Four times**
Blood sample prescription	Once	Once
Urine sample prescription	Once	Once
Counseling*	Two of four areas	All four areas
Experience of care		
Ability to discuss problems	"Yes"	"Yes"
Respectful care	"Yes"	"Yes"

*Areas of counseling: nutrition, breastfeeding, facility delivery and PP care
**IFA provision, weight and BP measurement are included at each visit in the FANC model. Other references do not indicate frequency of provision throughout pregnancy

Table 3. Characteristics of participants

Characteristic	Mean	SD	Range
Woman's age, years	22.5	4.2	16 to 41

For peer review only

Estimated GA at enrollment, months	3.5	1.3	1 to 9
Total number of visits per woman	4.5	2.5	1 to 14
	N	%	
SES quartiles*			
1	167	38%	
2	74	17%	
3	132	30%	
4	61	14%	
Did this woman have any prior live births?			
Yes	298	69%	
No	136	31%	
Did the woman have any years of education?			
No	259	60%	
Yes	175	40%	
Trimester at enrollment			
1-3 months	190	44%	
4-6 months	236	55%	
7-9 months	7	2%	
Total number of visits per woman			
1 visit	46	11%	
2-4 visits	205	47%	
5-7 visits	125	29%	
8+ visits	58	13%	
Number of visits during recommended months (<4, 4-6, 7-8, 9+)			
1	74	17%	
2	100	23%	
3	126	29%	
4	133	31%	

Table 4. Distribution of provision & experience of care during ANC

Provision of care	Never	Once	More than once
IFA or medicine-related			
Received IFA tablets	8.3%	19.8%	71.9%
Received a deworming	25.6%	73.5%	0.9%
Was given or told to buy calcium	13.8%	26.3%	59.9%
Physical exam			
Blood pressure was measured	2.8%	19.1%	78.1%
Weight measured	1.6%	17.1%	81.3%

For peer review only - http://bmjopen.bmj.com/site/about/guidelines.xhtml

Was given a prescription for a blood sample	6.7%	59.0%	34.3%
Was given a prescription for a urine sample	7.6%	55.5%	36.9%
Counseling			
Was told why deworming was being given	93.8%	6.2%	0.0%
Was told anything about blood pressure measurement	4.4%	21.0%	74.7%
Was told anything about weight gain or loss	2.1%	19.1%	78.8%
Was told why biospecimen (urine or blood) should be given	8.5%	42.9%	48.6%
Was given advice about food or nutrition during pregnancy	5.3%	19.4%	75.3%
Was told not drink and/or smoke	49.8%	27.9%	22.4%
Was given advice about breastfeeding	70.5%	7.1%	22.4%
Was told to deliver in a facility	28.3%	32.3%	39.4%
Was given advice on postpartum-related subjects	67.5%	11.8%	20.7%
Experience of care	N (%)		
Reported being able to discuss problems and concerns	379 (87.3%)		
Reported level of respect of treatment by health provider			
Very disrespectful	2 (0.5%)		
Somewhat disrespectful	2 (0.5%)		
Neither disrespectful nor respectful	5 (1.2%)		
Somewhat respectful	135 (31.0%)		
Very respectful	290 (66.8%)		

Table 5. Receipt by gestational age at visit

Service observed	Less than 4 months	4-6 months	7-8 months	9+ months	Chi-square p-value
IFA or medicine related					
Received IFA tablets	20.90%	66.40%	69.10%	69.00%	p <0.01
Woman was given deworming tablets	20.60%	30.50%	0.90%	0.00%	p <0.01
Woman was given or told to buy calcium tablets	19.70%	51.10%	53.20%	45.60%	p <0.01
Physical exam					

For peer review only - http://bmjopen.bmj.com/site/about/guidelines.xhtml

Woman's blood pressure was measured	69.60%	65.80%	67.50%	70.80%	0.335
Woman's weight was measured	69.00%	68.40%	68.20%	69.90%	0.958
Woman was given a prescription for a blood sample	56.40%	36.50%	16.60%	13.40%	p <0.01
Woman was given a prescription for a urine sample	56.10%	37.20%	17.30%	13.40%	p <0.01
Counseling					
Woman was told why deworming tablet was given	3.00%	1.60%	0.50%	0.60%	0.015
Woman was told anything about her BP	66.00%	61.20%	62.70%	63.80%	0.479
Woman was told anything about weight/gain/loss	67.50%	65.80%	64.10%	65.70%	0.805
Was told why biospecimen (urine or blood) should be given	53.40%	41.70%	25.80%	24.30%	p <0.01
Woman was given advice about food/nutrition during pregnancy	72.50%	64.00%	61.30%	60.20%	p <0.01
Received counseling on smoking and/or drinking	29.60%	22.30%	13.10%	16.70%	p <0.01
Woman was given any advice on breastfeeding	14.30%	12.10%	16.40%	28.90%	p <0.01
Woman was told to deliver in a facility	28.40%	25.60%	27.20%	61.10%	p <0.01
Received counseling on postpartum-related subjects	14.90%	13.00%	15.90%	28.60%	p <0.01

Table 6. Regression results

	Definition 1 (QOC1): Total study population (N=434)		Definition 2 (QOC4+): Among women with 4+ visits N=(262)	
	Bivariate model	Multivariate model	Bivariate model	Multivariate model
	RR (95% CI)	aRR (95% CI)	RR (95% CI)	aRR (95% CI)
Maternal age ≤ 20	1.14	1.27*	0.98	1.53

For peer review only - http://bmjopen.bmj.com/site/about/guidelines.xhtml

	(0.94-1.38)	(1.03-1.58)	(0.47-2.02)	(0.69-3.43)
No prior live birth	1.00 (0.82-1.24)	0.81 (0.64-1.02)	0.70 (0.31-1.60)	0.60 (0.23-1.56)
Any years of education	1.10 (0.91-1.34)	0.96 (0.80-1.16)	1.05 (0.51-2.16)	0.62 (0.31-1.26)
Number of visits	1.07** (1.04-1.11)	1.18** (1.13-1.23)	0.96 (0.80-1.15)	1.46** (1.11-2.80)
Enrollment during first trimester	1.03 (0.85-1.25)	1.22* (1.01-1.49)	1.07 (0.52-2.18)	1.15 (0.63-2.08)
SES quartiles (ref: 1st)				
2	1.27 (0.97-1.67)	1.16 (0.92-1.47)	0.67 (0.19-2.42)	1.77 (0.52-6.03)
3	1.21 (0.95-1.54)	0.99 (0.79-1.24)	1.77 (0.76-4.10)	1.48 (0.64-3.40)
4	1.31 (0.99-1.74)	1.09 (0.82-1.45)	1.22 (0.39-3.82)	1.17 (0.37-3.71)
Health post (ref: 1)				
2	0.80 (0.56-1.12)	0.66* (0.48-0.91)	---[1]	---[1]
3	1.62** (1.18-2.23)	1.69** (1.23-2.30)	24.73** (3.46-176.86)	36.11** (5.03-259.10)
4	1.63** (1.22-2.19)	1.84** (1.40-2.41)	7.68* (1.00-59.11)	12.59* (1.67-94.82)
5	1.55* (1.10-2.18)	1.95** (1.39-2.73)	2.82 (0.19-42.70)	6.22 (0.47-81.92)

*- $p<0.05$
**- $p<0.01$
[1]- There were no women who received good quality of care by the QOC4+ definition

For peer review only

Figure 1

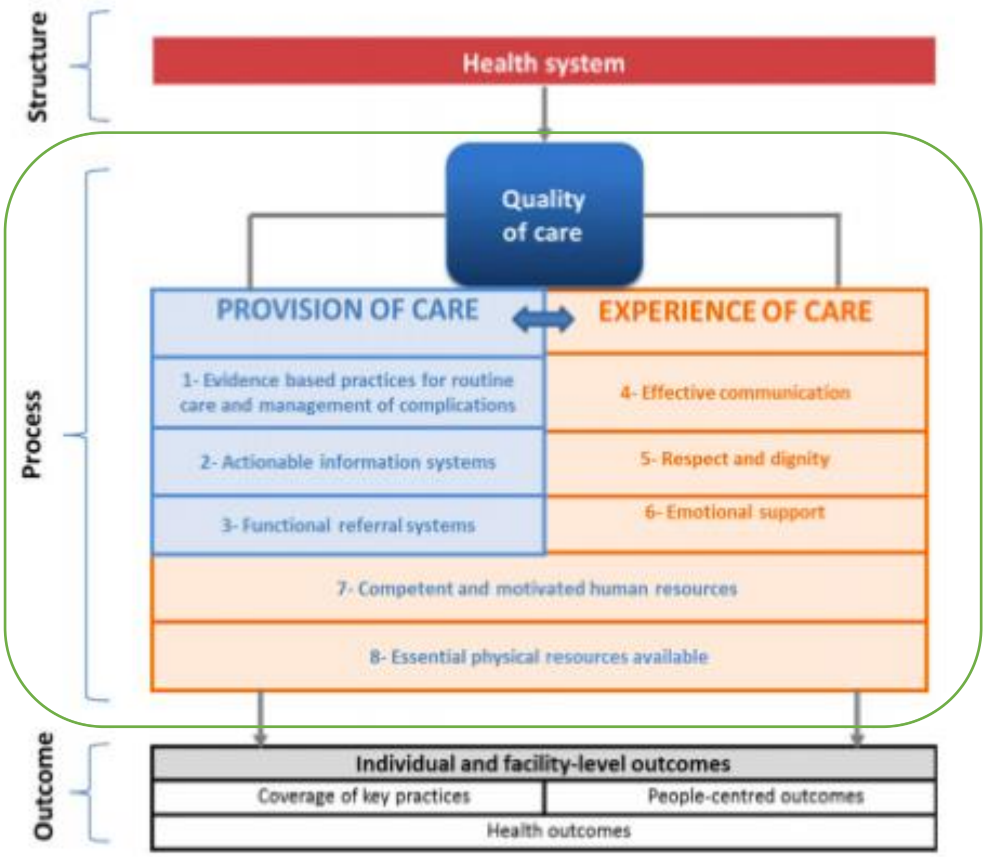
Structure
Health system
Process
Quality of care
PROVISION OF CARE
EXPERIENCE OF CARE
1- Evidence based practices for routine care and management of complications
2- Actionable information systems
3- Functional referral systems
4- Effective communication
5- Respect and dignity
6- Emotional support
7- Competent and motivated human resources
8- Essential physical resources available
Outcome
Individual and facility-level outcomes
Coverage of key practices
People-centred outcomes
Health outcomes

Figures 2a. QOC1 and 2b. QOC4+

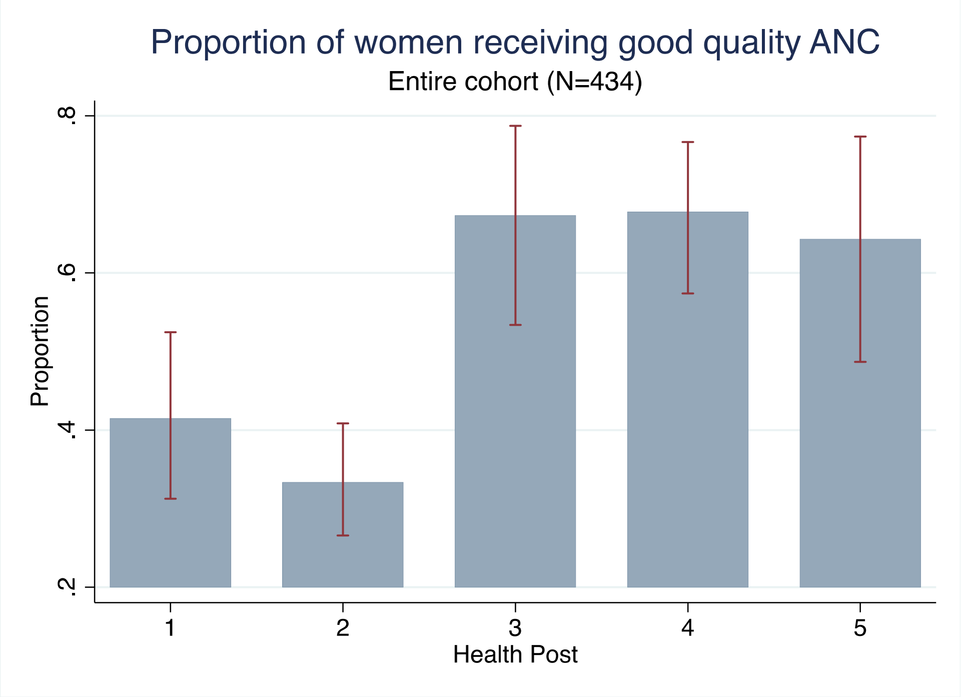

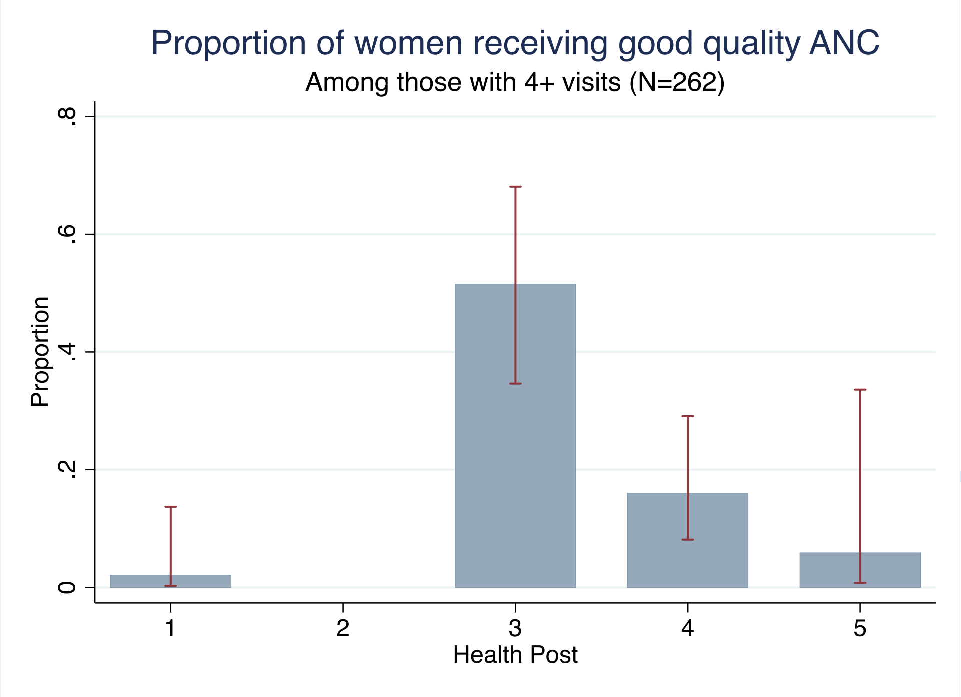

Figures 3a. QOC1

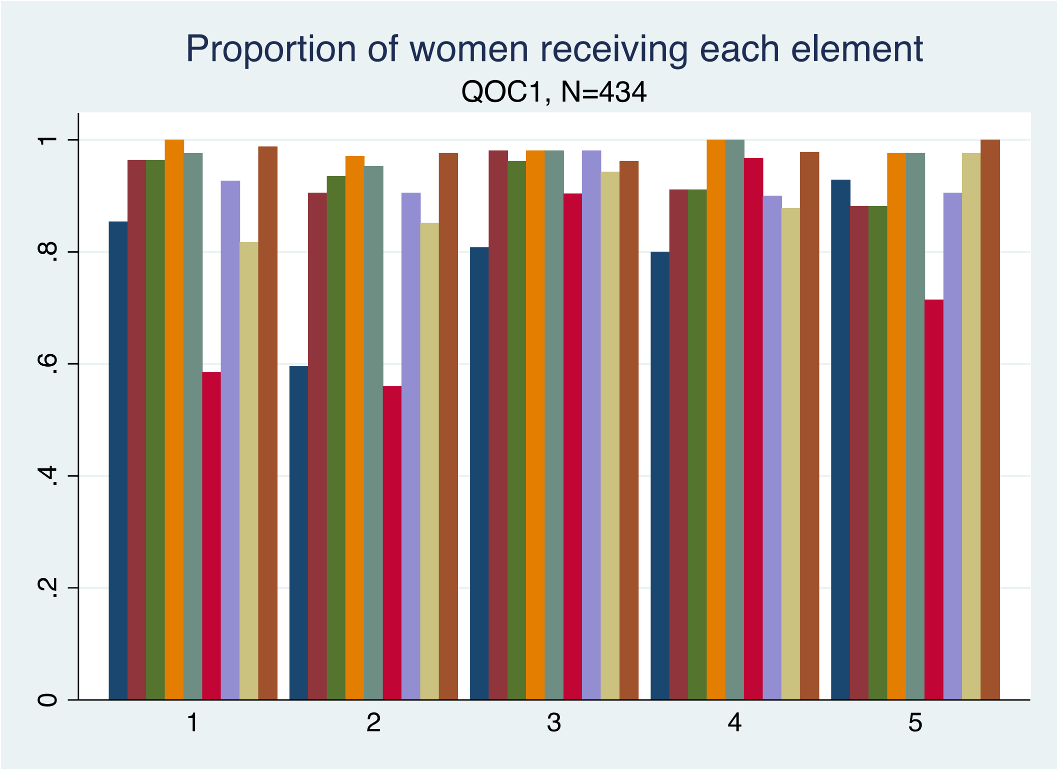

Figure 3b. QOC4+

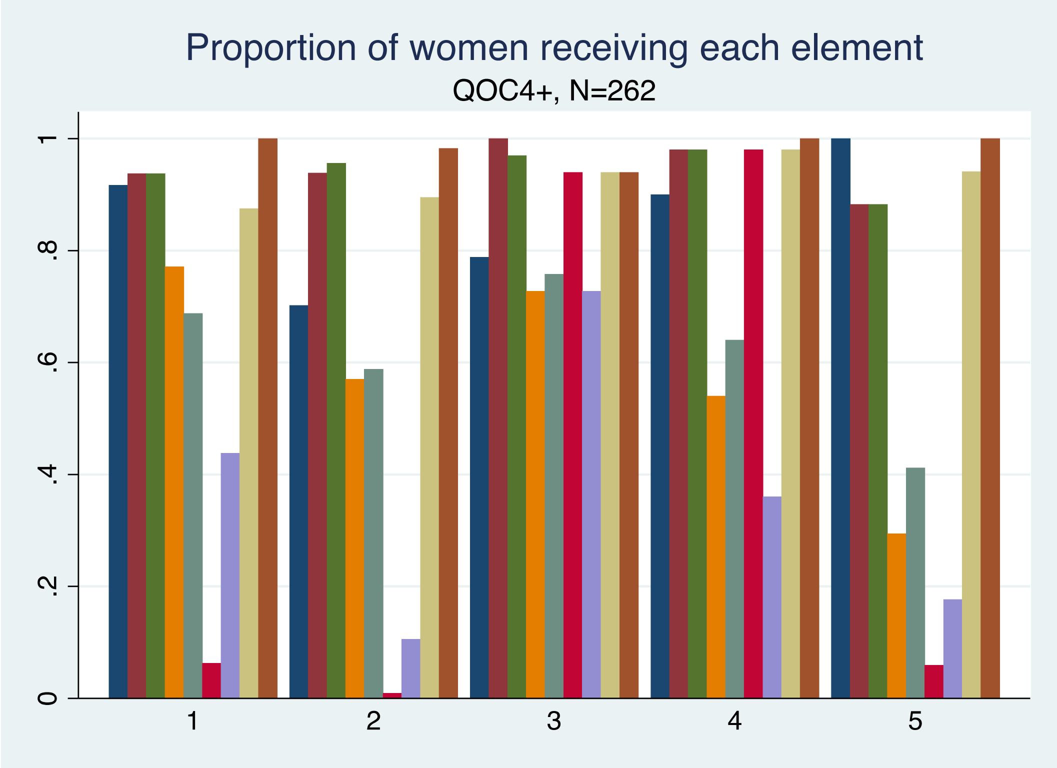

For peer review only

STROBE Statement—Checklist of items that should be included in reports of ***cohort studies***

	Item No	Recommendation	Page No
**Title and abstract**	1	(*a*) Indicate the study's design with a commonly used term in the title or the abstract<br>(*b*) Provide in the abstract an informative and balanced summary of what was done and what was found	1, 2
**Introduction**			
Background/rationale	2	Explain the scientific background and rationale for the investigation being reported	3-5
Objectives	3	State specific objectives, including any prespecified hypotheses	5
**Methods**			
Study design	4	Present key elements of study design early in the paper	5
Setting	5	Describe the setting, locations, and relevant dates, including periods of recruitment, exposure, follow-up, and data collection	5
Participants	6	(*a*) Give the eligibility criteria, and the sources and methods of selection of participants. Describe methods of follow-up<br>(*b*) For matched studies, give matching criteria and number of exposed and unexposed	5
Variables	7	Clearly define all outcomes, exposures, predictors, potential confounders, and effect modifiers. Give diagnostic criteria, if applicable	6
Data sources/ measurement	8*	For each variable of interest, give sources of data and details of methods of assessment (measurement). Describe comparability of assessment methods if there is more than one group	5-7
Bias	9	Describe any efforts to address potential sources of bias	11
Study size	10	Explain how the study size was arrived at	5
Quantitative variables	11	Explain how quantitative variables were handled in the analyses. If applicable, describe which groupings were chosen and why	6-7
Statistical methods	12	(*a*) Describe all statistical methods, including those used to control for confounding<br>(*b*) Describe any methods used to examine subgroups and interactions<br>(*c*) Explain how missing data were addressed<br>(*d*) If applicable, explain how loss to follow-up was addressed<br>(*e*) Describe any sensitivity analyses	7
**Results**			
Participants	13*	(a) Report numbers of individuals at each stage of study—eg numbers potentially eligible, examined for eligibility, confirmed eligible, included in the study, completing follow-up, and analysed<br>(b) Give reasons for non-participation at each stage<br>(c) Consider use of a flow diagram	7
Descriptive data	14*	(a) Give characteristics of study participants (eg demographic, clinical, social) and information on exposures and potential confounders<br>(b) Indicate number of participants with missing data for each variable of interest<br>(c) Summarise follow-up time (eg, average and total amount)	7-8
Outcome data	15*	Report numbers of outcome events or summary measures over time	8

For peer review only

For peer review only - http://bmjopen.bmj.com/site/about/guidelines.xhtml

Main results	16	(*a*) Give unadjusted estimates and, if applicable, confounder-adjusted estimates and their precision (eg, 95% confidence interval). Make clear which confounders were adjusted for and why they were included<br>(*b*) Report category boundaries when continuous variables were categorized<br>(*c*) If relevant, consider translating estimates of relative risk into absolute risk for a meaningful time period	8-9
Other analyses	17	Report other analyses done—eg analyses of subgroups and interactions, and sensitivity analyses	NA
**Discussion**			
Key results	18	Summarise key results with reference to study objectives	9
Limitations	19	Discuss limitations of the study, taking into account sources of potential bias or imprecision. Discuss both direction and magnitude of any potential bias	11
Interpretation	20	Give a cautious overall interpretation of results considering objectives, limitations, multiplicity of analyses, results from similar studies, and other relevant evidence	9-11
Generalisability	21	Discuss the generalisability (external validity) of the study results	12
**Other information**			
Funding	22	Give the source of funding and the role of the funders for the present study and, if applicable, for the original study on which the present article is based	1

*Give information separately for exposed and unexposed groups.

Note: An Explanation and Elaboration article discusses each checklist item and gives methodological background and published examples of transparent reporting. The STROBE checklist is best used in conjunction with this article (freely available on the Web sites of PLoS Medicine at http://www.plosmedicine.org/, Annals of Internal Medicine at http://www.annals.org/, and Epidemiology at http://www.epidem.com/). Information on the STROBE Initiative is available at http://www.strobe-statement.org.

For peer review only

BMJ Open

Antenatal care processes in rural Southern Nepal: gaps in and quality of service provision

Journal:	*BMJ Open*
Manuscript ID	bmjopen-2021-056392.R1
Article Type:	Original research
Date Submitted by the Author:	05-Nov-2021
Complete List of Authors:	Bryce, Emily; Johns Hopkins University Bloomberg School of Public Health,<br>Katz, Joanne; Johns Hopkins University Bloomberg School of Public Health, International Health<br>Plama, Tsering; Johns Hopkins University Bloomberg School of Public Health, Department of International Health<br>Khatry, Subarna; Nepal Nutr Intervent Project Sarlahi<br>LeClerq, Steven; Johns Hopkins University Bloomberg School of Public Health, International Health; Nepal Nutr Intervention Project, Sarlahi<br>Munos, Melinda; Johns Hopkins Bloomberg School of Public Health
**Primary Subject Heading**:	Public health
Secondary Subject Heading:	Global health
Keywords:	PUBLIC HEALTH, International health services < HEALTH SERVICES ADMINISTRATION & MANAGEMENT, Quality in health care < HEALTH SERVICES ADMINISTRATION & MANAGEMENT

SCHOLARONE™
Manuscripts

I, the Submitting Author has the right to grant and does grant on behalf of all authors of the Work (as defined in the below author licence), an exclusive licence and/or a non-exclusive licence for contributions from authors who are: i) UK Crown employees; ii) where BMJ has agreed a CC-BY licence shall apply, and/or iii) in accordance with the terms applicable for US Federal Government officers or employees acting as part of their official duties; on a worldwide, perpetual, irrevocable, royalty-free basis to BMJ Publishing Group Ltd ("BMJ") its licensees and where the relevant Journal is co-owned by BMJ to the co-owners of the Journal, to publish the Work in this journal and any other BMJ products and to exploit all rights, as set out in our licence.

The Submitting Author accepts and understands that any supply made under these terms is made by BMJ to the Submitting Author unless you are acting as an employee on behalf of your employer or a postgraduate student of an affiliated institution which is paying any applicable article publishing charge ("APC") for Open Access articles. Where the Submitting Author wishes to make the Work available on an Open Access basis (and intends to pay the relevant APC), the terms of reuse of such Open Access shall be governed by a Creative Commons licence – details of these licences and which Creative Commons licence will apply to this Work are set out in our licence referred to above.

Other than as permitted in any relevant BMJ Author's Self Archiving Policies, I confirm this Work has not been accepted for publication elsewhere, is not being considered for publication elsewhere and does not duplicate material already published. I confirm all authors consent to publication of this Work and authorise the granting of this licence.

For peer review only

Title: Antenatal care processes in rural Southern Nepal: gaps in and quality of service provision

Running Title: Quality of antenatal care in Southern Nepal

Authors: Emily Bryce, PhD[1], Joanne Katz, ScD[1] , Tsering Pema Lama, PhD[1,2], Subarna K. Khatry, MBBS, FRCS[2], Steven LeClerq, MPH[2] , Melinda Munos, PhD[1]

1-Johns Hopkins Bloomberg School of Public Health, Baltimore, Maryland USA 2-Nepal Nutrition Intervention Project, Sarlahi Nepal (NNIPS).

Corresponding author: Emily Bryce. 615 N Wolfe St, St Baltimore, MD 21205. (607) 592-5479. Email: ebryce2@jhu.edu

Word count: 4,242 (excluding headers and captions)
Number of references: 36
Number of Tables: 6
Number of Figures: 5

Conflict of interest: EB, MM, TL, SK, SLC and JK have no conflicts of interest.

Contribution statement: MM and JK designed the study. TL, SKK, SL, JK and EB contributed to the implementation of the study. EB conducted analyses and wrote the first draft of manuscript. MM and JK submitted edits to the manuscript. MM, JK, TL, SKK, EB and SL all have read and approve of the final version of the manuscript.

Funding: This research was funded by the Bill and Melinda Gates Foundation (grant number OPP1172551). The funders had no role in the design or implementation of the study, nor the analysis or interpretation of the data. JK and MM were the funding recipients

Data sharing: Data is available by emailing Joanne Katz, ScD or Emily Bryce, PhD

For peer review only

Abstract

Objective: This study aimed to compare a standard quality of care definition to one that reflected focused antenatal care (FANC) guidelines and examine associations with receipt of good quality of care.

Methods: In a longitudinal cohort study, we observed all the antenatal care visits 434 women attended at five government health posts in Southern Nepal. We examined differences in frequency of provision and service provision by gestational age at visit. We developed two quality of care definitions: 1) provision of seven services at least once during pregnancy and 2) provision of services to reflect the FANC guidelines by incorporating a frequency of care dimension for certain services. Multivariate log-binomial and Poisson with robust variance models were used to examine associations with receipt of good quality of care.

Findings: There was variation in service provision both in terms of frequency of provision and by gestational age at the visit. There were 213 women (49.1%) that received good quality care by the first definition, but when the frequency of service provision was included for the second definition the percentage dropped to 6.2%. There were significant differences in provision of quality care by health post. The number of visits, maternal age and care during the first trimester was associated with greater likelihood of good quality antenatal care.

Conclusion: This analysis demonstrated that measuring quality of care by receipt of services at least once during pregnancy may overestimate the true coverage of quality of antenatal care. Future efforts should improve feasibility of including frequency of care in quality of care definitions.

Strengths and limitations of this study

- Services received during antenatal care was established using direct observation rather than maternal report, thus eliminating the possibility the risk of recall bias
- Content of care was observed across the entire pregnancy, rather than cross sectionally at a single visit
- The study observers were required to meet inter and intra-observer reliability standards before study began
- Potential for observer effect, whereby providers altered their care because of the presence of the study observers

For peer review only

For peer review only - http://bmjopen.bmj.com/site/about/guidelines.xhtml

- Smaller sample size because of the resources required for the longitudinal nature of the study

For peer review only

Background

Despite the progress made in the previous two decades, in 2017 approximately 810 women died every day from preventable pregnancy and childbirth related causes.(1) The vast majority of these deaths occurred in low and lower-middle income countries. However, there are existing interventions that reduce maternal and neonatal mortality and morbidity that can be delivered during pregnancy.(2-5)

For peer review only - http://bmjopen.bmj.com/site/about/guidelines.xhtml

Along the reproductive health continuum, antenatal care (ANC) is defined as care provided during pregnancy by a skilled health provider, often at a first level facility.(3) ANC provides the opportunity to identify pregnancy risk factors through screening processes, prevent and manage diseases (pre-existing or pregnancy-related), and to provide health education and promotion.(3) Furthermore, good quality ANC has been associated with higher rates of facility delivery, which improves birth outcomes for mother and infant.(6, 7)

Guidelines for antenatal care have evolved over time, moving from a European model involving an average of twelve visits to the World Health Organization (WHO) four-visit, focused antenatal care model (FANC) in 2002 to the most current 2016 WHO guidelines requiring a minimum of eight visits during pregnancy.(3, 8) However, for many countries the transition to the eight-visit model has not yet been made. For example, Nepal's national protocol still follows the FANC model, which recommends four visits by four, six, eight and nine months gestational age.(9, 10) The country's safe motherhood program, *"Aama Suraksha"*, provides financial rewards to women who attended ANC during those four specified months and who deliver in a health facility.(9) Beyond the scheduling recommendations there are suggested components of ANC in Nepal, but there are no current, published guidelines; the last maternity guidelines were published in 1996.(11)

The coverage indicator ANC4+, or the proportion of pregnant women who attend four or more antenatal care visits, served as the measure of adequate antenatal care for the fifth Millennium Development Goal (MDG) and for countries to monitor their maternal care programs.(12) However, this indicator does not capture the content of the care provided, but rather contact with the health system. This is particularly problematic for ANC, as there are several interventions and health messages required for complete ANC.(3, 13, 14) Over the years, the measurement of ANC quality has shifted from contact-focused to content-focused.(12)

There is no single definition for "quality of care" for antenatal care, which complicates its measurement.(15) The WHO defines quality of care as healthcare that is "safe, effective, timely, efficient, equitable, and people-centered".(14) Accompanying this definition is a framework (Figure 1), which combines the Donabedian systems approach of evaluation (structure, process, outcome) and the two components of process quality (provision and experience of care) that are central components to other authors' definitions of quality of care.(14, 16-19)

In Nepal, 84% of women received any ANC from a skilled provider and 69% of women had at least four ANC visits, though the proportion is lower in rural areas.(20) 58.6% of women had a visit during the four months (4th, 6th, 8th and 9th) per national protocol. Although coverage of ANC has increased, as of 2016 the maternal mortality ratio was equal to 259 deaths per 100,000 live births, well above the SDG target of 70 deaths per 100,000 live births.(20) This indicates a gap in quality for provision of maternal care, including antenatal care.

For peer review only - http://bmjopen.bmj.com/site/about/guidelines.xhtml

A few other studies have examined the quality of antenatal care in Nepal and have shown significant gaps in quality of care, though some of the analyses use Demographic Health Survey data that is cross-sectional and subject to recall bias.(5, 21-23) To our knowledge, this is the first study in Nepal to use data generated by a direct observation during a longitudinal cohort study to examine antenatal care. Furthermore, the longitudinal nature of this study allows us to assess the frequency of service provision, as there are certain services (i.e. blood pressure and weight measurement) that should occur at every antenatal care visit. This study aims to describe the provision of antenatal care in our study population and to investigate factors associated with high quality ANC.

Methods

Data Sources

The data for this analysis were collected through the Coverage Validation Study, which was conducted within a part of the study area of the Nepal Nutrition Intervention Project Sarlahi (NNIPS) located in the Sarlahi District of rural, Southern Nepal. This study is one in a series of validation studies focused on measurement of nutrition-related indicators during pregnancy. The parent study aimed to enroll 450 women to end up with 300 women with complete antenatal care observation and completed follow-up interview for adequate power for the validation analyses. A total of 434 women were enrolled in the parent study, which represents the sample for this analysis.

Convenience sampling was used to enroll all eligible women as they presented at ANC at the five government health posts. The health posts were selected based on accessibility for the study team and client case load (15-25 patients per week). Pregnant, married women, ages 15 and older who were presenting for their first antenatal care visit at one of five government health posts selected for this study were eligible to participate. If a woman presented after already attending an ANC visit or ultrasound appointment, she was deemed ineligible because the study would not be able to observe all ANC visits.

A demographic questionnaire was administered at the enrollment visit. The direct observation of the first and all subsequent ANC visits at the study health posts were conducted by trained study staff, using a checklist of 28 items. The socioeconomic and client satisfaction data were collected during a postpartum interview.

Process quality of care assessment

The aim of this analysis was to evaluate the quality of the processes of care provided during antenatal care. The process of care includes two components, provision of care and experience of care (Figure 1).

For peer review only - http://bmjopen.bmj.com/site/about/guidelines.xhtml

Given that Nepal's national protocol is based on four antenatal visits, the FANC model served as the standard for the provision of care. In addition to the FANC model, Nepal's Ministry of Health (MoH) annual report, a country-led quality analysis of the 2016 DHS and an expert survey were reviewed to determine final service inclusion (Table 1).(22, 24-26) The MoH report and 2016 DHS were referenced to include the country's priorities and for comparison to other quality of care analyses. Additionally, the MoH annual report is the most recent documentation available with recommended antenatal care services in Nepal.

The provision of care domain was measured by receipt of the following: blood pressure measurement, weight measurement, IFA supplementation, blood test prescription, urine test prescription, deworming medication and counseling messages related to nutrition, breastfeeding, facility delivery and postpartum care. The prescription of blood and urine tests, rather than the performance of the test, was recorded because only one of the five health posts had a laboratory on-site for use. The parent study focused on nutrition-specific interventions, therefore we did not capture "counseling on complications and/or warning signs", which is commonly included in antenatal quality of care definitions (23, 27-29). Similarly, how the tetanus toxoid vaccine is distributed in this setting did not allow for its capture during observations and its inclusion in the quality of care metric.

We developed two quality of care definitions (Table 2). The process quality for the total study population is defined to align with other analyses of DHS or similarly collected cross-sectional data; at any point during a woman's pregnancy, she received any IFA (non-zero number of tablets), counseling in two of four areas (hypothesized to be dependent on gestational age), and the other five provision of care services at least once (QOC1). For the women who attended four or more ANC visits, quality was measured as at least 120 IFA tablets received, weight and BP measurements at least four times (as these services should be received at each ANC visit), counseling in all four areas and the remaining services at least once (QOC4+). Experience of care was measured the same in both groups.

Covariates

There were six maternal characteristics that were included to examine their association with quality of care: maternal age, maternal education, prior live births, trimester at enrollment household socioeconomic status and health post attendance. Maternal education was dichotomized in "zero years of education" and "any years of education" and prior live births into "no prior live births" and "one or more prior live birth". The trimester at enrollment was calculated by subtracting the date of reported last menstrual period (LMP) from the date of enrollment. Gestational age at each visit was calculated by subtracting the date of LMP from the visit date. These were then categorized into four groups (< 4 months, 4-6 months, 7-8 months and 9+ months) to reflect the FANC model groups. The household socioeconomic status was constructed

For peer review only - http://bmjopen.bmj.com/site/about/guidelines.xhtml

through the summation of 11 variables (including number of rooms, fuel and water sources, latrine type and ownership variables) and dividing the total sum by the total number of non-missing responses. This proportion was then separated into quartiles. Nine of the 434 women (2%) attended more than one health post during observations; the remaining 98% of participants attended the same health post for all observations. For those nine women, the health post they attended more frequently was considered their "primary health post".

Statistical analysis

Receipt of services was examined by gestational age using chi-square tests. Descriptive statistics were estimated to compare quality of care provided at each of the five health posts and between women of different characteristics using proportions for categorical variables and means for continuous variables.

Bivariable and multivariable log-binomial regression models were used to estimate risk ratios and the corresponding 95% confidence intervals for the associations between receipt of 1) QOC1 and 2) QOC4+. Relative risk estimates were calculated instead of odds ratios because the outcome was not rare (>10% of women received good quality of care) and therefore an odds ratio would have overestimated the relationships being examined If the log-binomial regression model did not converge, a poisson model with robust error variance was used. All analyses were conducted using Stata version 14.2 (Stat Corp, College Station, Texas).

Patient and Public Involvement

Study participants were not involved in this design, recruitment, conduct or dissemination of this research. The 28-item checklist was reviewed by a local community advisory board in Nepal before the start of the study, but the public had no other part in the development or implementation of this study. There are no plans to disseminate results to the participants or community, aside from the local study staff who reside in the community.

Ethical approval statement

The Institutional Review Board of the Johns Hopkins Bloomberg School of Public Health (Baltimore, Maryland) and the National Health Research Council (Kathmandu, Nepal) approved this study. Informed consent was obtained from providers and participants at the beginning of the study and participants were re-consented at the postpartum interview.

Results

For peer review only - http://bmjopen.bmj.com/site/about/guidelines.xhtml

A total of 441 women were enrolled in the study and seven women (1.6% of the participants) were lost to follow-up for the six-month postpartum interview, resulting in a final sample of 434 women. The women were between 16 and 41 years old at enrollment, with an average age of 22.5 years (Table 3). Sixty percent had 4 or more ANC visits and 31% of women attended a visit during each of the four recommended months outlined by the FANC model. Sixty percent of the women reported no years of education and 32% had no prior live births.

Frequency of an intervention or counseling message being observed, categorized as never, once or more than once and the reported experience of care is shown in Table 4. The majority of IFA and medication-related interventions were received once or more than once. Four women (0.9%) were observed receiving deworming medication twice, which is outside recommendations. The majority of women had blood pressure and weight measured more than once (78.1% and 81.3%, respectively). Over 70% of women did not receive any counseling regarding breastfeeding and 67.5% never received advice on postpartum related subjects (e.g., importance of postpartum visits, when to come for the visit). Eighty-seven percent of women reported being able to discuss problems and concerns with their provider and 97.8% reported somewhat or very respectful care.

There were no differences in the measurement of or advice concerning blood pressure or weight by gestational age at the visit (Table 5). The proportion of women receiving the service in each of the four categories was significantly different for all other services. A greater proportion of women beyond four months gestational age received IFA tablets and were given or told to buy calcium tablets than less than four months. Conversely, a higher proportion of women received deworming medication and prescriptions for blood and urine tests at visits that occurred at less than four months and between four to six months. Nutrition-related advice and counseling on ceasing smoking and drinking was more frequently provided at visits earlier in pregnancy. Advice on matters more related to delivery (breastfeeding, delivery in a facility) and postpartum subjects were given to a higher proportion of women at nine months gestational age or greater.

Quality of care

There were 213 women (49.1%) of the total 434 that were observed receiving good quality of antenatal care by the first definition (QOC1). The proportion significantly decreased to 10.3% (27 of 262 women) when the second definition was applied among women with four or more visits (QOC4+); 6.2% of the entire cohort. There was significant variation between health posts and the proportion of women receiving good quality of care (Figures 2a & 2b). The second health post, at which the greatest number of women were enrolled, did not have any women with four or more visits receive good quality of care. The proportion of women that received each element of the quality of care definitions by health post are presented in Figures 3a and 3b to illustrate gaps in provision. Across the majority of the health posts, the element with the lowest

For peer review only - http://bmjopen.bmj.com/site/about/guidelines.xhtml

prevalence was the receipt of the appropriate number of counseling messages. While any IFA supplementation for QOC1 is not a limiting factor, the proportion of women who received the requisite 120+ tablets for QOC4+ is much lower across all health posts.

Table 6 presents the bivariate and multivariate regression results. The greater the number of ANC visits a woman attended was associated with improved quality of care by both definitions (QOC1 aRR= 1.18 [1.13-2.13, p<0.01]; QOC4+ aRR= 1.46 [1.11-2.80, p<0.01]). Additionally, women who were 20 years old or younger had 27% greater likelihood of receiving QOC1 than older women. Women who attended their first ANC visit during the first trimester were 22% more likely to have received QOC1. The direction and magnitude of these two covariate's associations with QOC4+ were similar to QOC1, but they were not statistically significant.

Discussion

This paper examined the process and quality of antenatal care in Southern Nepal using data from a longitudinal study of direct antenatal care observations. The analyses reveal there are gaps in provision of care and differences in services received by gestational age at visit. Nearly half of women received good quality of antenatal care defined by receipt of the services at least once during pregnancy and a good experience of care. However, when the QOC definition was expanded to reflect the FANC model recommendations, the percent drops to 6.2% of women receiving good quality of care. While over 60% of the women attended at least four ANC visits, only 31% attended a visit during each of the four recommended gestational age periods as outlined by the FANC model. This illustrates that aside from not capturing the content of care, the ANC4+ indicator may a poor measure of the recommended contacts for measuring antenatal care coverage.

The results show a gap in care for counseling during ANC, specifically messaging pertaining to breastfeeding and postpartum visits. Low rates of counseling in these areas have been documented in other studies of quality of antenatal care as well.(27, 29, 30) The high rate of counseling about nutrition in our study is similar to another in Nepal using DHS data.(23) The lower rates of counseling on facility delivery, breastfeeding and postpartum visits in our setting could be because the receipt of financial incentives for the safe motherhood program is contingent on facility delivery and it is assumed these messages will be delivered at that time. However, rates of facility delivery in Nepal are still quite low, 56% in 2016,(20) therefore these aspects of counseling should also be covered during antenatal care. Additionally, a recent review also found major gaps in quality of counseling messaging in Nepal and four other South Asian countries.(31) We did not measure the quality of the counseling messaging themselves, only their delivery. However, Torlesse et al.'s findings may mean that the coverage measures in our population may overestimate the proportion of women who received quality counseling that led to information transfer from provider to client.

Aside from weight and blood pressure measurement, which should be completed at every visit, there were significant differences in the proportion of women receiving a

For peer review only - http://bmjopen.bmj.com/site/about/guidelines.xhtml

service at a given gestational age. This corresponds with findings from studies in Pakistan and Bangladesh.(28, 32) The timing is a critical component of care because certain screening tests (i.e., blood test for syphilis) and IFA supplementation should be received earlier in pregnancy.(3) Furthermore, this has implications for women who do not attend ANC during all recommended months and may miss components of care.

The definition of quality of antenatal care is commonly the receipt of certain services at any point during pregnancy. Our data shows that using this definition, 49.1% of the women in our population received good quality of antenatal care. This is higher than another study using 2011 Nepal DHS data,(23) and studies conducted India, Bangladesh and in sub-Saharan Africa.(27, 28, 33, 34) A possible explanation could be that as our data is more recent, health service delivery has improved since the previous studies. Additionally, the other studies primarily rely on maternal recall of services received during ANC, many of which have yet to be validated, which could lead to reporting errors. Finally, our coverage of QOC1 could be higher due to the observer effect (discussed in full in limitations), resulting in the providers at the study clinics providing more complete care than they would if not under observation.

To our knowledge, this is the first study in a low-and-middle-income setting to examine quality of care by including frequency of service provision as outlined by the FANC model. A study in Kenya asked women to report whether they had their weight and blood pressure measured "never, a few times, most of the time and all the time" and found the majority of women to have received the two services most or all of the time.(29) This is comparable to our findings, but this study did not create a final QOC metric to which we can compare our QOC4+ results.

Our analysis showed that the proportion of women received good quality of care differed by health post, more so for QOC4+ than for QOC1. A multi-level model with a random intercept for health post and inclusion of health post level covariates would have been ideal for further elucidating reasons for these differences. However, with only five health posts, the number of clusters was too small to conduct these analyses. Exploring associations between provider characteristics and quality of care would be helpful to explain differences in provision, however the parent study did not collect this information.

The total number of ANC visits is the only characteristic that is significantly associated with both of the QOC definitions, which is consistent with another study.(33) The greater the number of visits a woman attended, the greater the opportunity to have received all necessary components. Younger women (<20 years) were more likely to have received better quality of care, which was not the case in other studies in Nepal and Kenya.(23, 29)

There were no differences in QOC by SES quartile in our study, whereas others have shown there to be a significant relationship between SES and QOC.(23, 33, 35) However, these studies primarily use data from population-based surveys (e.g., DHS) and have a more heterogeneous population in regard to SES. In our population, the

For peer review only - http://bmjopen.bmj.com/site/about/guidelines.xhtml

mean score was 6.3 (SD=2.3) and was skewed towards lower scores. There was no difference in QOC1 by education, which has been demonstrated in some studies (29, 36) but not others.(23, 27, 33) As with socioeconomic status, our population was more homogenous in educational attainment; 60% of the population reported no years of education and only one woman reported schooling beyond grade school, compared to 38.9% with no education and 7% with tertiary education in the 2011 Nepal DHS study.(23)

There were no statistically significant associations between covariates and QOC4+, which could be due to a lack of power (discussed further in limitations), as the magnitude of the coefficient for many is quite large. While we cannot draw conclusions on associations between the covariates and the QOC4+ outcome, the descriptive analyses demonstrate the gaps in care and difference in coverage between QOC4+ and QOC1. The health post with the greatest number of patients had no women with high quality QOC4+, driven primarily by a lack of counseling. This health post also had the smallest proportion of women who received 120+ tablets and deworming medication. The increased patient load could explain the low proportions of women receiving the commodity-based services; stock outs might have occurred, or the providers may have limited the number of IFA tablets given to a woman at any one visit to delay a stockout. The lack of counseling could be due to time limitations; the provider may feel that they cannot spend adequate time to convey all the health education messaging because of waiting patients. However, the satisfaction score for this health post was high and similar to the other posts.

A strength of this study is that the receipt of services was collected by direct observation, rather than maternal report. The use of direct observation removes the possibility for recall bias and social desirability bias from the participant, both of which can be present in the cross-sectional studies commonly used in antenatal quality of care studies. Furthermore, the longitudinal design of the parent study allowed for the observation of a woman's entire pregnancy and record of frequency of service provision. Another strength includes the use of trained observers who were required to meet inter and intra-observer reliability standards before the study began.

There are limitations to this analysis as well. While the data is not subject to recall or social desirability bias, the presence of the study observers during ANC could have elicited an observer effect, where the providers knew they were being observed and altered their care. To reduce this possibility, providers were told during the consent process that observers are not medically trained and will not know if the procedures they are observing are correct or not, nor will they report what they observe to the clinic supervisor. Additionally, the observers were present for over a year and one would hope that any observer effect would dissipate over the extended time period. The client satisfaction data was collected at the postpartum interview, which means it is subject to recall and social desirability bias. However, there has been evidence of "courtesy bias", where individuals are less likely to report critically at the facility, which is avoided in this case. Additionally, gestational age estimation was based on maternal report of last menstrual period, which is subject to recall bias as well. Eighty-one percent of the

For peer review only - http://bmjopen.bmj.com/site/about/guidelines.xhtml

sample presented by the fourth month of pregnancy, so the hope is that with a shorter recall period the LMP data is still fairly accurate. Another limitation was that we only observed women who presented for antenatal care at government health facilities. Therefore, the generalizability may be limited to women who attend antenatal care or those who remain within the system in which we observed (excluding private facilities, traditional healers, etc,). A final limitation is the smaller sample size compared other ANC QOC studies, which may have led to limited power in detecting associations between QOC and maternal and facility characteristics. However, because the longitudinal direct observation design is so time and resource intensive, it would be difficult to get a much larger sample size.

This analysis demonstrates that measuring quality of care by receipt of services at least once during pregnancy may be overestimating the true coverage of quality of antenatal care. Furthermore, as countries transition to the eight-visit 2016 WHO ANC recommendations, the degree of underestimation will only increase. Additionally, despite a common belief that commodity-based services such as IFA tablets are often the limiting factor for quality of care, in this study the largest gap in service provision was seen for adequate counseling messaging and the counseling adequacy varied significantly across the study health posts. Future programming and policy efforts should fortify provider training on effective counseling delivery and interpersonal skills to close the gap in counseling provision. This analysis supports the continued research and programmatic efforts in strengthening of routine health information systems and electronic health records to measure quality of care over a woman's entire pregnancy. This would allow the frequency of service provision to be included in quality of care assessment. Additionally, these systems and household surveys should be updated to capture additional indicators of counseling delivery. The inclusion of frequency of specific services provided in ANC in quality of care measurement may be the next step in closing the "quality-coverage" gap and achieving optimal maternal, newborn and child survival.

Conflict of interest: EB, MM, TPL, SK, SLC and JK have no conflicts of interest.

Contribution statement: MM and JK designed the study. TPL, SKK, SL, JK and EB contributed to the implementation of the study. EB conducted analyses and wrote the first draft of manuscript. MM and JK submitted edits to the manuscript. MM, JK, TPL, SKK, EB and SL all have read and approve of the final version of the manuscript.

Funding: This research was funded by the Bill and Melinda Gates Foundation (grant number OPP1172551). The funders had no role in the design or implementation of the study, nor the analysis or interpretation of the data. JK and MM were the funding recipients

Data sharing: Data is available by emailing Joanne Katz, ScD or Emily Bryce, PhD

For peer review only - http://bmjopen.bmj.com/site/about/guidelines.xhtml

Figure legends/caption:

Figure 1 caption: *Source: Tuncalp, Ö et al. Quality of care for pregnant women and newborns-the WHO vision(14)*

Figure 2a and 2b: N/A

Figure 3a legend:

	Deworming		Urine sample
	Blood sample		Weight
	Blood Pressure		2+ counseling
	Any IFA		Discuss problems
	Respectful care		

Figure 3b legend:

	Deworming		Urine sample
	Blood sample		Weight
	Blood Pressure		≥ 4 counseling
	≥ 120 IFA		Discuss problems
	Respectful care		

References

1. Trends in maternal mortality 2000 to 2017: estimates by WHO, UNICEF, UNFPA, World Bank Group and the United Nations Population Division. Geneva: World Health Organization; 2019.

2. Carroli G, Rooney C, Villar J. How effective is antenatal care in preventing maternal mortality and serious morbidity? An overview of the evidence. Paediatric and perinatal epidemiology. 2001;15 Suppl 1:1-42.

3. WHO. WHO recommendations on antenatal care for a positive pregnancy experience. Geneva, Switzerland; 2016.

4. Kuhnt J, Vollmer S. Antenatal care services and its implications for vital and health outcomes of children: evidence from 193 surveys in 69 low-income and middle-income countries. BMJ Open. 2017;7(11):e017122.

5. Neupane S, Nwaru BI. Impact of prenatal care utilization on infant care practices in Nepal: a national representative cross-sectional survey. European journal of pediatrics. 2014;173(1):99-109.

For peer review only

6. Guliani H, Sepehri A, Serieux J. What impact does contact with the prenatal care system have on women's use of facility delivery? Evidence from low-income countries. Soc Sci Med. 2012;74(12):1882-90.
7. Bloom SS, Lippeveld T, Wypij D. Does antenatal care make a difference to safe delivery? A study in urban Uttar Pradesh, India. Health Policy Plan. 1999;14(1):38-48.
8. Villar J, Ba'aqeel H, Piaggio G, et al. WHO antenatal care randomised trial for the evaluation of a new model of routine antenatal care. Lancet (London, England). 2001;357(9268):1551-64.
9. Pandey S. Women's knowledge about the conditional cash incentive program and its association with institutional delivery in Nepal. PloS one. 2018;13(6):e0199230.
10. WHO. WHO antenatal care randomized trial: manual for the implementation of the new model. Geneva; 2002.
11. Jahn A, Dar Iang M, Shah U, et al. Maternity care in rural Nepal: a health service analysis. Tropical medicine & international health : TM & IH. 2000;5(9):657-65.
12. Hodgins S, D'Agostino A. The quality-coverage gap in antenatal care: toward better measurement of effective coverage. Glob Health Sci Pract. 2014;2(2):173-81.
13. Bhutta ZA, Darmstadt GL, Hasan BS, et al. Community-based interventions for improving perinatal and neonatal health outcomes in developing countries: a review of the evidence. Pediatrics. 2005;115(2 Suppl):519-617.
14. Tuncalp, Were WM, MacLennan C, et al. Quality of care for pregnant women and newborns-the WHO vision. BJOG : an international journal of obstetrics and gynaecology. 2015;122(8):1045-9.
15. Raven JH, Tolhurst RJ, Tang S, et al. What is quality in maternal and neonatal health care? Midwifery. 2012;28(5):e676-83.
16. Donabedian A. Exploratings in quality assessment and monitoring definition of quality and approaches to its assessment. 1980.
17. Donabedian A. The quality of care. How can it be assessed? Jama. 1988;260(12):1743-8.
18. Hulton L, Matthews Z, Stones RW. A framework for the evaluation of quality of care in maternity services. 2000.
19. Bruce J. Fundamental elements of the quality of care: a simple framework. Stud Fam Plann. 1990;21(2):61-91.
20. Nepal MoH. Nepal Demographic and Health Survey 2016. Kathmandu, Nepal: Ministry of Health, Nepal; 2017.
21. Aryal K, Sharad SK, Mukti NK, Bihungum B, Shiv SL, Shambhu K, and Mona SM. Maternal Health Care in Nepal: Trends and Determinants. Rockville, Maryland, USA: ICF; 2019.
22. Lama TP, M. Chaulagain, A. Rai, and S. Gurung. Assessment of the Quality of Antenatal Care, Family Planning, and Sick Child Care Services in Nepal: Further Analysis of the 2015 Nepal Health Facility Survey. Rockville, Maryland, USA: ICF; 2019.
23. Joshi C, Torvaldsen S, Hodgson R, et al. Factors associated with the use and quality of antenatal care in Nepal: a population-based study using the demographic and health survey data. BMC pregnancy and childbirth. 2014;14:94.
24. Population MoHa. Annual Report, Department of Health Services 2074/2075 (2017/2018). Kathmandu, Nepal; 2019.

For peer review only

25. Population MoHa. Annual Report, Department of Health Services 2070/71 (2013/2014). Kathmandu, Nepal; 2015.
26. Sheffel A, Zeger S, Heidkamp R, et al. Development of summary indices of antenatal care service quality in Haiti, Malawi and Tanzania. BMJ Open. 2019;9(12):e032558.
27. Islam MM, Masud MS. Determinants of frequency and contents of antenatal care visits in Bangladesh: Assessing the extent of compliance with the WHO recommendations. PloS one. 2018;13(9):e0204752.
28. Siddique AB, Perkins J, Mazumder T, et al. Antenatal care in rural Bangladesh: Gaps in adequate coverage and content. PloS one. 2018;13(11):e0205149.
29. Afulani PA, Buback L, Essandoh F, et al. Quality of antenatal care and associated factors in a rural county in Kenya: an assessment of service provision and experience dimensions. BMC Health Serv Res. 2019;19(1):684.
30. Marchant T, Tilley-Gyado RD, Tessema T, et al. Adding content to contacts: measurement of high quality contacts for maternal and newborn health in Ethiopia, north east Nigeria, and Uttar Pradesh, India. PloS one. 2015;10(5):e0126840.
31. Andrews RM, McCarthy J, Carapetis JR, et al. Skin Disorders, Including Pyoderma, Scabies, and Tinea Infections. Pediatric Clinics of North America. 2009;56(6):1421-40.
32. Agha S, Tappis H. The timing of antenatal care initiation and the content of care in Sindh, Pakistan. BMC pregnancy and childbirth. 2016;16(1):190.
33. Olorunsaiye CZ, Brunner Huber LR, Laditka SB, et al. Individual and community socioeconomic factors related to the quality of antenatal care: a multilevel analysis of West and Central Africa. Women Health. 2021;61(1):15-26.
34. Singh L, Dubey R, Singh S, et al. Measuring quality of antenatal care: a secondary analysis of national survey data from India. BJOG : an international journal of obstetrics and gynaecology. 2019;126 Suppl 4:7-13.
35. Sharma J, Leslie HH, Kundu F, et al. Poor Quality for Poor Women? Inequities in the Quality of Antenatal and Delivery Care in Kenya. PloS one. 2017;12(1):e0171236.
36. Lee E, Madhavan S, Bauhoff S. Levels and variations in the quality of facility-based antenatal care in Kenya: evidence from the 2010 service provision assessment. Health Policy Plan. 2016;31(6):777-84.

Table 1

	FANC	Nepal MoH annual report	Nepal DHS analyses	Expert survey	What CVS-2 captured
Provision of care					
IFA	X	X	X	X	X
IPTp	X			(not for Nepal)	
TT vaccine	X	X	X	X	

Deworming		X	X	X	X
BP measured	X	X (2017 report)	X	X	X
Weight measured			X		X
Blood sample	X		X	X	X
Urine sample	X		X	X	X
Counseling	X	X	X	X	X
Nutrition	X	X	X	X	X
Breastfeeding	X			X	X
Facility delivery	X	X	X		X
Family planning			X		
PP care		X			X
Experience of care					
Ability to discuss problems			X	X	X
Respectful care			X	X	X
Satisfied with explanation of information			X	X	

Table 2. Quality of care definitions

	Definition 1: Entire study population (QOC1)	Definition 2: Women who had recommended 4+ visits (QOC4+)
Provision of care		
IFA	Any	120+ (30/visit)**
Deworming	Once	Once
BP measured	Once	Four times**
Weight measured	Once	Four times**
Blood sample prescription	Once	Once
Urine sample prescription	Once	Once
Counseling*	Two of four areas	All four areas
Experience of care		
Ability to discuss problems	"Yes"	"Yes"
Respectful care	"Yes"	"Yes"

*Areas of counseling: nutrition, breastfeeding, facility delivery and PP care
**IFA provision, weight and BP measurement are included at each visit in the FANC model. Other references do not indicate frequency of provision throughout pregnancy

For peer review only

For peer review only - http://bmjopen.bmj.com/site/about/guidelines.xhtml

Table 3. Characteristics of participants

Characteristic	Mean	SD	Range
Woman's age, years	22.5	4.2	16 to 41
Estimated GA at enrollment, months	3.5	1.3	1 to 9
Total number of visits per woman	4.5	2.5	1 to 14
	N	%	
SES quartiles*			
1	167	38%	
2	74	17%	
3	132	30%	
4	61	14%	
Did this woman have any prior live births?			
Yes	298	69%	
No	136	31%	
Did the woman have any years of education?			
No	259	60%	
Yes	175	40%	
Trimester at enrollment			
1-3 months	190	44%	
4-6 months	236	55%	
7-9 months	7	2%	
Total number of visits per woman			
1 visit	46	11%	
2-4 visits	205	47%	
5-7 visits	125	29%	
8+ visits	58	13%	
Number of visits during recommended months (<4, 4-6, 7-8, 9+)			
1	74	17%	
2	100	23%	
3	126	29%	
4	133	31%	

Table 4. Distribution of provision & experience of care during ANC

Provision of care	Never	Once	More than once
IFA or medicine-related			
Received IFA tablets	8.3%	19.8%	71.9%
Received a deworming	25.6%	73.5%	0.9%
Was given or told to buy calcium	13.8%	26.3%	59.9%
Physical exam			

For peer review only - http://bmjopen.bmj.com/site/about/guidelines.xhtml

Blood pressure was measured	2.8%	19.1%	78.1%
Weight measured	1.6%	17.1%	81.3%
Was given a prescription for a blood sample	6.7%	59.0%	34.3%
Was given a prescription for a urine sample	7.6%	55.5%	36.9%
Counseling			
Was told why deworming was being given	93.8%	6.2%	0.0%
Was told anything about blood pressure measurement	4.4%	21.0%	74.7%
Was told anything about weight gain or loss	2.1%	19.1%	78.8%
Was told why biospecimen (urine or blood) should be given	8.5%	42.9%	48.6%
Was given advice about food or nutrition during pregnancy	5.3%	19.4%	75.3%
Was told not drink and/or smoke	49.8%	27.9%	22.4%
Was given advice about breastfeeding	70.5%	7.1%	22.4%
Was told to deliver in a facility	28.3%	32.3%	39.4%
Was given advice on postpartum-related subjects	67.5%	11.8%	20.7%
Experience of care	N (%)		
Reported being able to discuss problems and concerns	379 (87.3%)		
Reported level of respect of treatment by health provider			
Very disrespectful	2 (0.5%)		
Somewhat disrespectful	2 (0.5%)		
Neither disrespectful nor respectful	5 (1.2%)		
Somewhat respectful	135 (31.0%)		
Very respectful	290 (66.8%)		

Table 5. Receipt by gestational age at visit

Service observed	Less than 4 months	4-6 months	7-8 months	9+ months	Chi-square p-value
IFA or medicine related					
Received IFA tablets	20.90%	66.40%	69.10%	69.00%	p <0.01
Woman was given deworming tablets	20.60%	30.50%	0.90%	0.00%	p <0.01

For peer review only - http://bmjopen.bmj.com/site/about/guidelines.xhtml

Woman was given or told to buy calcium tablets	19.70%	51.10%	53.20%	45.60%	p <0.01
Physical exam					
Woman's blood pressure was measured	69.60%	65.80%	67.50%	70.80%	0.335
Woman's weight was measured	69.00%	68.40%	68.20%	69.90%	0.958
Woman was given a prescription for a blood sample	56.40%	36.50%	16.60%	13.40%	p <0.01
Woman was given a prescription for a urine sample	56.10%	37.20%	17.30%	13.40%	p <0.01
Counseling					
Woman was told why deworming tablet was given	3.00%	1.60%	0.50%	0.60%	0.015
Woman was told anything about her BP	66.00%	61.20%	62.70%	63.80%	0.479
Woman was told anything about weight/gain/loss	67.50%	65.80%	64.10%	65.70%	0.805
Was told why biospecimen (urine or blood) should be given	53.40%	41.70%	25.80%	24.30%	p <0.01
Woman was given advice about food/nutrition during pregnancy	72.50%	64.00%	61.30%	60.20%	p <0.01
Received counseling on smoking and/or drinking	29.60%	22.30%	13.10%	16.70%	p <0.01
Woman was given any advice on breastfeeding	14.30%	12.10%	16.40%	28.90%	p <0.01
Woman was told to deliver in a facility	28.40%	25.60%	27.20%	61.10%	p <0.01
Received counseling on postpartum-related subjects	14.90%	13.00%	15.90%	28.60%	p <0.01

Table 6. Regression results

	Definition 1 (QOC1): Total study population (N=434)	Definition 2 (QOC4+): Among women with 4+ visits N=(262)

For peer review only - http://bmjopen.bmj.com/site/about/guidelines.xhtml

	Bivariate model	Multivariate model	Bivariate model	Multivariate model
	RR (95% CI)	aRR (95% CI)	RR (95% CI)	aRR (95% CI)
Maternal age ≤ 20	1.14 (0.94-1.38)	1.27* (1.03-1.58)	0.98 (0.47-2.02)	1.53 (0.69-3.43)
No prior live birth	1.00 (0.82-1.24)	0.81 (0.64-1.02)	0.70 (0.31-1.60)	0.60 (0.23-1.56)
Any years of education	1.10 (0.91-1.34)	0.96 (0.80-1.16)	1.05 (0.51-2.16)	0.62 (0.31-1.26)
Number of visits	1.07** (1.04-1.11)	1.18** (1.13-1.23)	0.96 (0.80-1.15)	1.46** (1.11-2.80)
Enrollment during first trimester	1.03 (0.85-1.25)	1.22* (1.01-1.49)	1.07 (0.52-2.18)	1.15 (0.63-2.08)
SES quartiles (ref: 1st)				
2	1.27 (0.97-1.67)	1.16 (0.92-1.47)	0.67 (0.19-2.42)	1.77 (0.52-6.03)
3	1.21 (0.95-1.54)	0.99 (0.79-1.24)	1.77 (0.76-4.10)	1.48 (0.64-3.40)
4	1.31 (0.99-1.74)	1.09 (0.82-1.45)	1.22 (0.39-3.82)	1.17 (0.37-3.71)
Health post (ref: 1)				
2	0.80 (0.56-1.12)	0.66* (0.48-0.91)	---[1]	---[1]
3	1.62** (1.18-2.23)	1.69** (1.23-2.30)	24.73** (3.46-176.86)	36.11** (5.03-259.10)
4	1.63** (1.22-2.19)	1.84** (1.40-2.41)	7.68* (1.00-59.11)	12.59* (1.67-94.82)
5	1.55* (1.10-2.18)	1.95** (1.39-2.73)	2.82 (0.19-42.70)	6.22 (0.47-81.92)

*- p<0.05
**- p<0.01
[1]- There were no women who received good quality of care by the QOC4+ definition

For peer review only - http://bmjopen.bmj.com/site/about/guidelines.xhtml

Figure 1

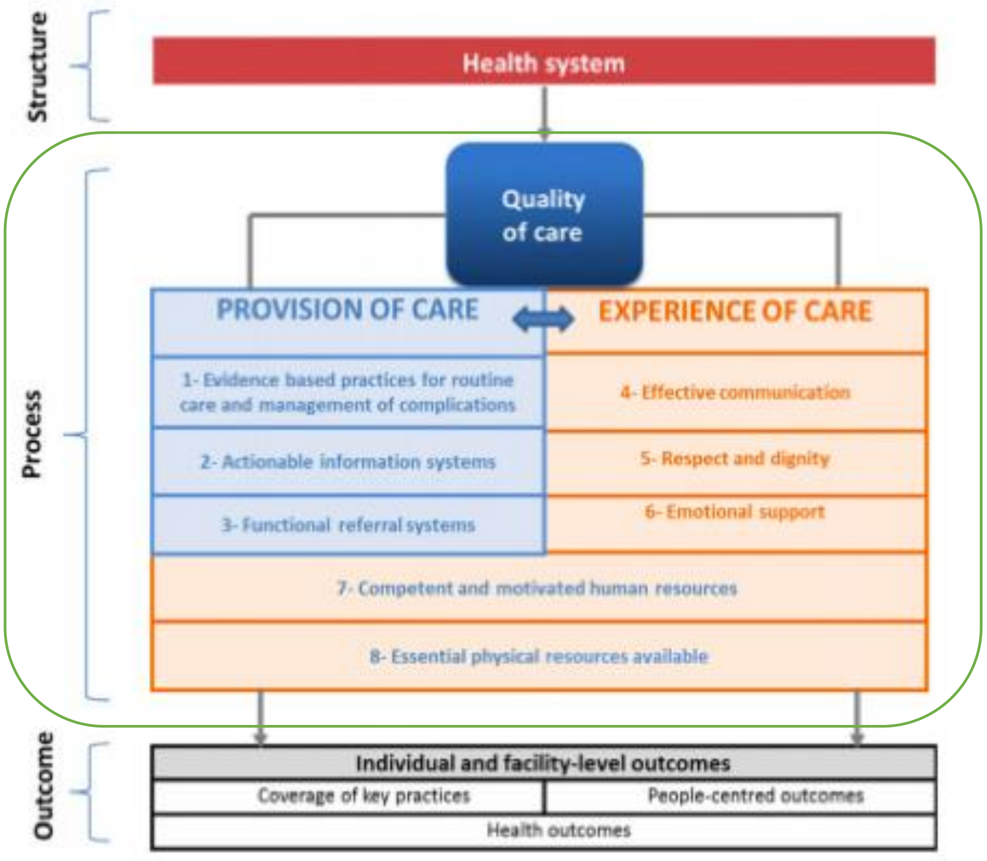

For peer review only - http://bmjopen.bmj.com/site/about/guidelines.xhtml

Figures 2a. QOC1 and 2b. QOC4+

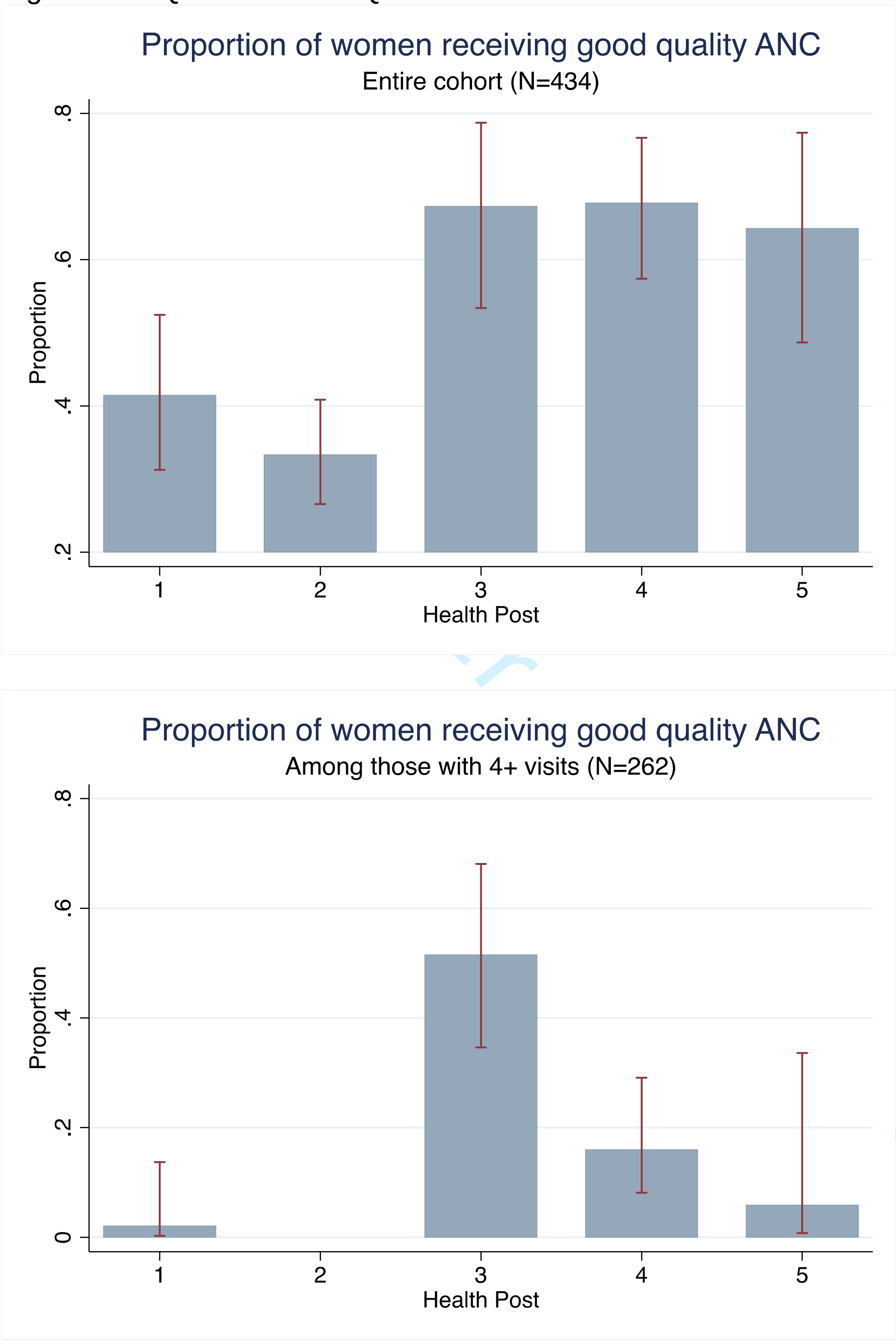

Figures 3a. QOC1

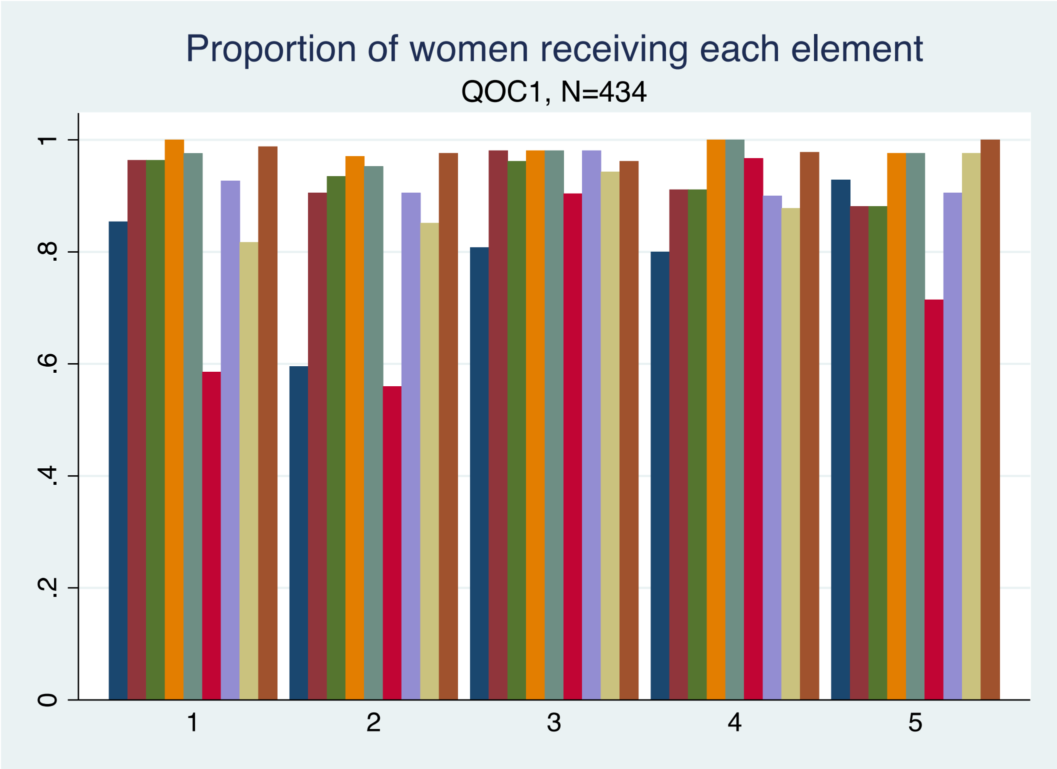

Figure 3b. QOC4+

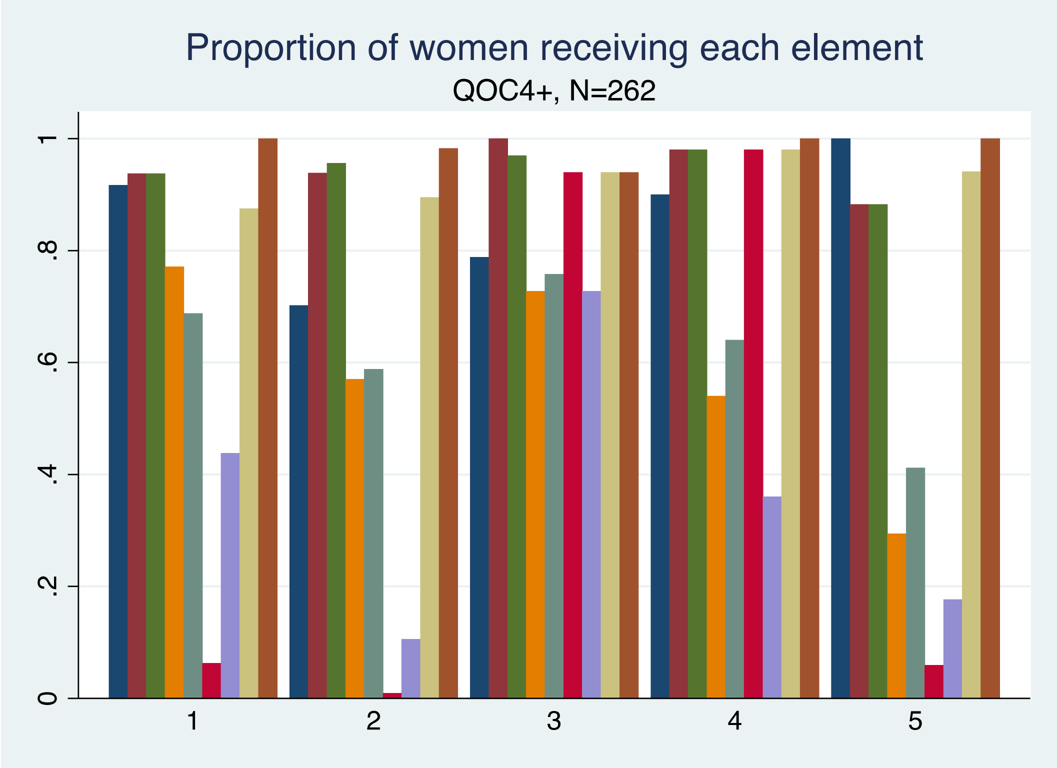

For peer review only

STROBE Statement—Checklist of items that should be included in reports of ***cohort studies***

	Item No	Recommendation	Page No
**Title and abstract**	1	(*a*) Indicate the study's design with a commonly used term in the title or the abstract<br>(*b*) Provide in the abstract an informative and balanced summary of what was done and what was found	1, 2
**Introduction**			
Background/rationale	2	Explain the scientific background and rationale for the investigation being reported	3-5
Objectives	3	State specific objectives, including any prespecified hypotheses	5
**Methods**			
Study design	4	Present key elements of study design early in the paper	5
Setting	5	Describe the setting, locations, and relevant dates, including periods of recruitment, exposure, follow-up, and data collection	5
Participants	6	(*a*) Give the eligibility criteria, and the sources and methods of selection of participants. Describe methods of follow-up<br>(*b*) For matched studies, give matching criteria and number of exposed and unexposed	5
Variables	7	Clearly define all outcomes, exposures, predictors, potential confounders, and effect modifiers. Give diagnostic criteria, if applicable	6
Data sources/ measurement	8*	For each variable of interest, give sources of data and details of methods of assessment (measurement). Describe comparability of assessment methods if there is more than one group	5-7
Bias	9	Describe any efforts to address potential sources of bias	11
Study size	10	Explain how the study size was arrived at	5
Quantitative variables	11	Explain how quantitative variables were handled in the analyses. If applicable, describe which groupings were chosen and why	6-7
Statistical methods	12	(*a*) Describe all statistical methods, including those used to control for confounding<br>(*b*) Describe any methods used to examine subgroups and interactions<br>(*c*) Explain how missing data were addressed<br>(*d*) If applicable, explain how loss to follow-up was addressed<br>(*e*) Describe any sensitivity analyses	7
**Results**			
Participants	13*	(a) Report numbers of individuals at each stage of study—eg numbers potentially eligible, examined for eligibility, confirmed eligible, included in the study, completing follow-up, and analysed<br>(b) Give reasons for non-participation at each stage<br>(c) Consider use of a flow diagram	7
Descriptive data	14*	(a) Give characteristics of study participants (eg demographic, clinical, social) and information on exposures and potential confounders<br>(b) Indicate number of participants with missing data for each variable of interest<br>(c) Summarise follow-up time (eg, average and total amount)	7-8
Outcome data	15*	Report numbers of outcome events or summary measures over time	8

For peer review only

Main results	16	(*a*) Give unadjusted estimates and, if applicable, confounder-adjusted estimates and their precision (eg, 95% confidence interval). Make clear which confounders were adjusted for and why they were included<br>(*b*) Report category boundaries when continuous variables were categorized<br>(*c*) If relevant, consider translating estimates of relative risk into absolute risk for a meaningful time period	8-9
Other analyses	17	Report other analyses done—eg analyses of subgroups and interactions, and sensitivity analyses	NA
**Discussion**			
Key results	18	Summarise key results with reference to study objectives	9
Limitations	19	Discuss limitations of the study, taking into account sources of potential bias or imprecision. Discuss both direction and magnitude of any potential bias	11
Interpretation	20	Give a cautious overall interpretation of results considering objectives, limitations, multiplicity of analyses, results from similar studies, and other relevant evidence	9-11
Generalisability	21	Discuss the generalisability (external validity) of the study results	12
**Other information**			
Funding	22	Give the source of funding and the role of the funders for the present study and, if applicable, for the original study on which the present article is based	1

*Give information separately for exposed and unexposed groups.

Note: An Explanation and Elaboration article discusses each checklist item and gives methodological background and published examples of transparent reporting. The STROBE checklist is best used in conjunction with this article (freely available on the Web sites of PLoS Medicine at http://www.plosmedicine.org/, Annals of Internal Medicine at http://www.annals.org/, and Epidemiology at http://www.epidem.com/). Information on the STROBE Initiative is available at http://www.strobe-statement.org.

For peer review only

Antenatal care processes in rural Southern Nepal: gaps in and quality of service provision: a cohort study

Journal:	*BMJ Open*
Manuscript ID	bmjopen-2021-056392.R2
Article Type:	Original research
Date Submitted by the Author:	22-Nov-2021
Complete List of Authors:	Bryce, Emily; Johns Hopkins University Bloomberg School of Public Health,<br>Katz, Joanne; Johns Hopkins University Bloomberg School of Public Health, International Health<br>Plama, Tsering; Johns Hopkins University Bloomberg School of Public Health, Department of International Health<br>Khatry, Subarna; Nepal Nutr Intervent Project Sarlahi<br>LeClerq, Steven; Johns Hopkins University Bloomberg School of Public Health, International Health; Nepal Nutr Intervention Project, Sarlahi<br>Munos, Melinda; Johns Hopkins Bloomberg School of Public Health
**Primary Subject Heading**:	Public health
Secondary Subject Heading:	Global health
Keywords:	PUBLIC HEALTH, International health services < HEALTH SERVICES ADMINISTRATION & MANAGEMENT, Quality in health care < HEALTH SERVICES ADMINISTRATION & MANAGEMENT

SCHOLARONE™
Manuscripts

I, the Submitting Author has the right to grant and does grant on behalf of all authors of the Work (as defined in the below author licence), an exclusive licence and/or a non-exclusive licence for contributions from authors who are: i) UK Crown employees; ii) where BMJ has agreed a CC-BY licence shall apply, and/or iii) in accordance with the terms applicable for US Federal Government officers or employees acting as part of their official duties; on a worldwide, perpetual, irrevocable, royalty-free basis to BMJ Publishing Group Ltd ("BMJ") its licensees and where the relevant Journal is co-owned by BMJ to the co-owners of the Journal, to publish the Work in this journal and any other BMJ products and to exploit all rights, as set out in our licence.

The Submitting Author accepts and understands that any supply made under these terms is made by BMJ to the Submitting Author unless you are acting as an employee on behalf of your employer or a postgraduate student of an affiliated institution which is paying any applicable article publishing charge ("APC") for Open Access articles. Where the Submitting Author wishes to make the Work available on an Open Access basis (and intends to pay the relevant APC), the terms of reuse of such Open Access shall be governed by a Creative Commons licence – details of these licences and which Creative Commons licence will apply to this Work are set out in our licence referred to above.

Other than as permitted in any relevant BMJ Author's Self Archiving Policies, I confirm this Work has not been accepted for publication elsewhere, is not being considered for publication elsewhere and does not duplicate material already published. I confirm all authors consent to publication of this Work and authorise the granting of this licence.

For peer review only

Title: Antenatal care processes in rural Southern Nepal: gaps in and quality of service provision: a cohort study

Running Title: Quality of antenatal care in Southern Nepal

Authors: Emily Bryce, PhD[1], Joanne Katz, ScD[1] , Tsering Pema Lama, PhD[1,2], Subarna K. Khatry, MBBS, FRCS[2], Steven LeClerq, MPH[2] , Melinda Munos, PhD[1]

1-Johns Hopkins Bloomberg School of Public Health, Baltimore, Maryland USA
2- Nepal Nutrition Intervention Project, Sarlahi Nepal (NNIPS).

Corresponding author: Emily Bryce. 615 N Wolfe St, St Baltimore, MD 21205. (607) 592-5479. Email: ebryce2@jhu.edu

Word count: 4,299 (excluding headers and captions)
Number of references: 36
Number of Tables: 6
Number of Figures: 5

Conflict of interest: EB, MM, TPL, SK, SLC and JK have no conflicts of interest.

Contribution statement: MM and JK designed the study. TPL, SKK, SL, JK and EB contributed to the implementation of the study. EB conducted analyses and wrote the first draft of manuscript. MM and JK submitted edits to the manuscript. MM, JK, TPL, SKK, EB and SL all have read and approve of the final version of the manuscript.

Funding: This research was funded by the Bill and Melinda Gates Foundation (grant number OPP1172551). The funders had no role in the design or implementation of the study, nor the analysis or interpretation of the data. JK and MM were the funding recipients

Data sharing: Data is available by emailing Joanne Katz, ScD or Emily Bryce, PhD

For peer review only

Abstract

Objectives: This study aimed to compare a standard quality of care definition to one that reflected focused antenatal care (FANC) guidelines and examine associations with receipt of good quality of care.

Design: This study was a longitudinal cohort study

Settings: Five government health posts in the Sarlahi district of Southern Nepal

Participants: Pregnant women between the ages of 15 and 49 who presented for their first antenatal care visit at the study health posts.

Main outcomes: There were two quality of care definitions: 1) provision of seven services at least once during pregnancy (QOC1) and 2) provision of services to reflect the FANC guidelines by incorporating a frequency of care dimension for certain services (QOC4+)

Results: There was variation in service provision both in terms of frequency of provision and by gestational age at the visit. There were 213 women (49.1%) that received good quality care by the first definition, but when the frequency of service provision was included for the second definition the percentage dropped to 6.2%. There were significant differences in provision of quality care by health post for both definitions. The number of visits (QOC1 aRR 1.18, 95% CI 1.13 to 1.23, QOC4+ aRR 1.46, 95% CI 1.11 to 2.80)) and care during the first trimester (QOC1 aRR 1.22, 95% CI 1.01 to 1.49) and maternal age (QOC1 aRR 1.27, 95% CI 1.03 to 1.58) were associated with greater likelihood of good quality antenatal care.

Conclusion: This analysis demonstrated that measuring quality of care by receipt of services at least once during pregnancy may overestimate the true coverage of quality of antenatal care. Future efforts should improve feasibility of including frequency of care in quality of care definitions.

For peer review only

Strengths and limitations of this study

- Services received during antenatal care was established using direct observation rather than maternal report, thus eliminating the possibility the risk of recall bias
- Content of care was observed across the entire pregnancy, rather than cross sectionally at a single visit
- The study observers were required to meet inter and intra-observer reliability standards before study began

- Potential for observer effect, whereby providers altered their care because of the presence of the study observers
- Smaller sample size because of the resources required for the longitudinal nature of the study

For peer review only

Background

Despite the progress made in the previous two decades, in 2017 approximately 810 women died every day from preventable pregnancy and childbirth related causes.(1) The vast majority of these deaths occurred in low and lower-middle income

countries. However, there are existing interventions that reduce maternal and neonatal mortality and morbidity that can be delivered during pregnancy.(2-5)

Along the reproductive health continuum, antenatal care (ANC) is defined as care provided during pregnancy by a skilled health provider, often at a first level facility.(3) ANC provides the opportunity to identify pregnancy risk factors through screening processes, prevent and manage diseases (pre-existing or pregnancy-related), and to provide health education and promotion.(3) Furthermore, good quality ANC has been associated with higher rates of facility delivery, which improves birth outcomes for mother and infant.(6, 7)

Guidelines for antenatal care have evolved over time, moving from a European model involving an average of twelve visits to the World Health Organization (WHO) four-visit, focused antenatal care model (FANC) in 2002 to the most current 2016 WHO guidelines requiring a minimum of eight visits during pregnancy.(3, 8) However, for many countries the transition to the eight-visit model has not yet been made. For example, Nepal's national protocol still follows the FANC model, which recommends four visits by four, six, eight and nine months gestational age.(9, 10) The country's safe motherhood program, *"Aama Suraksha"*, provides financial rewards to women who attended ANC during those four specified months and who deliver in a health facility.(9) Beyond the scheduling recommendations there are suggested components of ANC in Nepal, but there are no current, published guidelines; the last maternity guidelines were published in 1996.(11)

The coverage indicator ANC4+, or the proportion of pregnant women who attend four or more antenatal care visits, served as the measure of adequate antenatal care for the fifth Millennium Development Goal (MDG) and for countries to monitor their maternal care programs.(12) However, this indicator does not capture the content of the care provided, but rather contact with the health system. This is particularly problematic for ANC, as there are several interventions and health messages required for complete ANC.(3, 13, 14) Over the years, the measurement of ANC quality has shifted from contact-focused to content-focused.(12)

There is no single definition for "quality of care" for antenatal care, which complicates its measurement.(15) The WHO defines quality of care as healthcare that is "safe, effective, timely, efficient, equitable, and people-centered".(14) Accompanying this definition is a framework (Figure 1), which combines the Donabedian systems approach of evaluation (structure, process, outcome) and the two components of process quality (provision and experience of care) that are central components to other authors' definitions of quality of care.(14, 16-19)

In Nepal, 84% of women received any ANC from a skilled provider and 69% of women had at least four ANC visits, though the proportion is lower in rural areas.(20) 58.6% of women had a visit during the four months (4th, 6th, 8th and 9th) per national protocol. Although coverage of ANC has increased, as of 2016 the maternal mortality

For peer review only - http://bmjopen.bmj.com/site/about/guidelines.xhtml

ratio was equal to 259 deaths per 100,000 live births, well above the SDG target of 70 deaths per 100,000 live births.(20) This indicates a gap in quality for provision of maternal care, including antenatal care.

A few other studies have examined the quality of antenatal care in Nepal and have shown significant gaps in quality of care, though some of the analyses use Demographic Health Survey data that is cross-sectional and subject to recall bias.(5, 21-23) To our knowledge, this is the first study in Nepal to use data generated by a direct observation during a longitudinal cohort study to examine antenatal care. Furthermore, the longitudinal nature of this study allows us to assess the frequency of service provision, as there are certain services (i.e. blood pressure and weight measurement) that should occur at every antenatal care visit. This study aims to describe the provision of antenatal care in our study population and to investigate factors associated with high quality ANC.

Methods

Data Sources

The data for this analysis were collected through the Coverage Validation Study, which was conducted within a part of the study area of the Nepal Nutrition Intervention Project Sarlahi (NNIPS) located in the Sarlahi District of rural, Southern Nepal. This study is one in a series of validation studies focused on measurement of nutrition-related indicators during pregnancy. The parent study aimed to enroll 450 women to end up with 300 women with complete antenatal care observation and completed follow-up interview for adequate power for the validation analyses. A total of 434 women were enrolled in the parent study, which represents the sample for this analysis.

Convenience sampling was used to enroll all eligible women as they presented at ANC at the five government health posts. The health posts were selected based on accessibility for the study team and client case load (15-25 patients per week). Pregnant, married women, ages 15 and older who were presenting for their first antenatal care visit at one of five government health posts selected for this study were eligible to participate. If a woman presented after already attending an ANC visit or ultrasound appointment, she was deemed ineligible because the study would not be able to observe all ANC visits.

A demographic questionnaire was administered at the enrollment visit. The direct observation of the first and all subsequent ANC visits at the study health posts were conducted by trained study staff, using a checklist of 28 items. The socioeconomic and client satisfaction data were collected during a postpartum interview.

Process quality of care assessment

For peer review only - http://bmjopen.bmj.com/site/about/guidelines.xhtml

The aim of this analysis was to evaluate the quality of the processes of care provided during antenatal care. The process of care includes two components, provision of care and experience of care (Figure 1).

Given that Nepal's national protocol is based on four antenatal visits, the FANC model served as the standard for the provision of care. In addition to the FANC model, Nepal's Ministry of Health (MoH) annual report, a country-led quality analysis of the 2016 DHS and an expert survey were reviewed to determine final service inclusion (Table 1).(22, 24-26) The MoH report and 2016 DHS were referenced to include the country's priorities and for comparison to other quality of care analyses. Additionally, the MoH annual report is the most recent documentation available with recommended antenatal care services in Nepal.

The provision of care domain was measured by receipt of the following: blood pressure measurement, weight measurement, IFA supplementation, blood test prescription, urine test prescription, deworming medication and counseling messages related to nutrition, breastfeeding, facility delivery and postpartum care. The prescription of blood and urine tests, rather than the performance of the test, was recorded because only one of the five health posts had a laboratory on-site for use. The parent study focused on nutrition-specific interventions, therefore we did not capture "counseling on complications and/or warning signs", which is commonly included in antenatal quality of care definitions (23, 27-29). Similarly, how the tetanus toxoid vaccine is distributed in this setting did not allow for its capture during observations and its inclusion in the quality of care metric.

We developed two quality of care definitions (Table 2). The process quality for the total study population is defined to align with other analyses of DHS or similarly collected cross-sectional data; at any point during a woman's pregnancy, she received any IFA (non-zero number of tablets), counseling in two of four areas (hypothesized to be dependent on gestational age), and the other five provision of care services at least once (QOC1). For the women who attended four or more ANC visits, quality was measured as at least 120 IFA tablets received, weight and BP measurements at least four times (as these services should be received at each ANC visit), counseling in all four areas and the remaining services at least once (QOC4+). Experience of care was measured the same in both groups.

Covariates

There were six maternal characteristics that were included to examine their association with quality of care: maternal age, maternal education, prior live births, trimester at enrollment household socioeconomic status and health post attendance. Maternal education was dichotomized in "zero years of education" and "any years of education" and prior live births into "no prior live births" and "one or more prior live birth". The trimester at enrollment was calculated by subtracting the date of reported last

For peer review only - http://bmjopen.bmj.com/site/about/guidelines.xhtml

menstrual period (LMP) from the date of enrollment. Gestational age at each visit was calculated by subtracting the date of LMP from the visit date. These were then categorized into four groups (< 4 months, 4-6 months, 7-8 months and 9+ months) to reflect the FANC model groups. The household socioeconomic status was constructed through the summation of 11 variables (including number of rooms, fuel and water sources, latrine type and ownership variables) and dividing the total sum by the total number of non-missing responses. This proportion was then separated into quartiles. Nine of the 434 women (2%) attended more than one health post during observations; the remaining 98% of participants attended the same health post for all observations. For those nine women, the health post they attended more frequently was considered their “primary health post”.

Statistical analysis

Receipt of services was examined by gestational age using chi-square tests. Descriptive statistics were estimated to compare quality of care provided at each of the five health posts and between women of different characteristics using proportions for categorical variables and means for continuous variables.

Bivariable and multivariable log-binomial regression models were used to estimate risk ratios and the corresponding 95% confidence intervals for the associations between receipt of 1) QOC1 and 2) QOC4+. Relative risk estimates were calculated instead of odds ratios because the outcome was not rare (>10% of women received good quality of care) and therefore an odds ratio would have overestimated the relationships being examined If the log-binomial regression model did not converge, a poisson model with robust error variance was used. All analyses were conducted using Stata version 14.2 (Stat Corp, College Station, Texas).

Patient and Public Involvement

Study participants were not involved in this design, recruitment, conduct or dissemination of this research. The 28-item checklist was reviewed by a local community advisory board in Nepal before the start of the study, but the public had no other part in the development or implementation of this study. There are no plans to disseminate results to the participants or community, aside from the local study staff who reside in the community.

Ethical approval statement

The Institutional Review Board of the Johns Hopkins Bloomberg School of Public Health (Baltimore, Maryland) and the National Health Research Council (Kathmandu, Nepal) approved this study. Informed consent was obtained from providers and participants at the beginning of the study and participants were re-consented at the postpartum interview.

For peer review only - http://bmjopen.bmj.com/site/about/guidelines.xhtml

Results

A total of 441 women were enrolled in the study and seven women (1.6% of the participants) were lost to follow-up for the six-month postpartum interview, resulting in a final sample of 434 women. The women were between 16 and 41 years old at enrollment, with an average age of 22.5 years (Table 3). Sixty percent had 4 or more ANC visits and 31% of women attended a visit during each of the four recommended months outlined by the FANC model. Sixty percent of the women reported no years of education and 32% had no prior live births.

Frequency of an intervention or counseling message being observed, categorized as never, once or more than once and the reported experience of care is shown in Table 4. The majority of IFA and medication-related interventions were received once or more than once. Four women (0.9%) were observed receiving deworming medication twice, which is outside recommendations. The majority of women had blood pressure and weight measured more than once (78.1% and 81.3%, respectively). Over 70% of women did not receive any counseling regarding breastfeeding and 67.5% never received advice on postpartum related subjects (e.g., importance of postpartum visits, when to come for the visit). Eighty-seven percent of women reported being able to discuss problems and concerns with their provider and 97.8% reported somewhat or very respectful care.

There were no differences in the measurement of or advice concerning blood pressure or weight by gestational age at the visit (Table 5). The proportion of women receiving the service in each of the four categories was significantly different for all other services. A greater proportion of women beyond four months gestational age received IFA tablets and were given or told to buy calcium tablets than less than four months. Conversely, a higher proportion of women received deworming medication and prescriptions for blood and urine tests at visits that occurred at less than four months and between four to six months. Nutrition-related advice and counseling on ceasing smoking and drinking was more frequently provided at visits earlier in pregnancy. Advice on matters more related to delivery (breastfeeding, delivery in a facility) and postpartum subjects were given to a higher proportion of women at nine months gestational age or greater.

Quality of care

There were 213 women (49.1%) of the total 434 that were observed receiving good quality of antenatal care by the first definition (QOC1). The proportion significantly decreased to 10.3% (27 of 262 women) when the second definition was applied among women with four or more visits (QOC4+); 6.2% of the entire cohort. There was significant variation between health posts and the proportion of women receiving good quality of care (Figures 2a & 2b). The second health post, at which the greatest number of women were enrolled, did not have any women with four or more visits receive good

For peer review only - http://bmjopen.bmj.com/site/about/guidelines.xhtml

quality of care. The proportion of women that received each element of the quality of care definitions by health post are presented in Figures 3a and 3b to illustrate gaps in provision. Across the majority of the health posts, the element with the lowest prevalence was the receipt of the appropriate number of counseling messages. While any IFA supplementation for QOC1 is not a limiting factor, the proportion of women who received the requisite 120+ tablets for QOC4+ is much lower across all health posts.

Table 6 presents the bivariate and multivariate regression results. The greater the number of ANC visits a woman attended was associated with improved quality of care by both definitions (QOC1 aRR= 1.18 [1.13-2.13, p<0.01]; QOC4+ aRR= 1.46 [1.11-2.80, p<0.01]). Additionally, women who were 20 years old or younger had 27% greater likelihood of receiving QOC1 than older women. Women who attended their first ANC visit during the first trimester were 22% more likely to have received QOC1. The direction and magnitude of these two covariate's associations with QOC4+ were similar to QOC1, but they were not statistically significant.

Discussion

This paper examined the process and quality of antenatal care in Southern Nepal using data from a longitudinal study of direct antenatal care observations. The analyses reveal there are gaps in provision of care and differences in services received by gestational age at visit. Nearly half of women received good quality of antenatal care defined by receipt of the services at least once during pregnancy and a good experience of care. However, when the QOC definition was expanded to reflect the FANC model recommendations, the percent drops to 6.2% of women receiving good quality of care. While over 60% of the women attended at least four ANC visits, only 31% attended a visit during each of the four recommended gestational age periods as outlined by the FANC model. This illustrates that aside from not capturing the content of care, the ANC4+ indicator may a poor measure of the recommended contacts for measuring antenatal care coverage.

The results show a gap in care for counseling during ANC, specifically messaging pertaining to breastfeeding and postpartum visits. Low rates of counseling in these areas have been documented in other studies of quality of antenatal care as well.(27, 29, 30) The high rate of counseling about nutrition in our study is similar to another in Nepal using DHS data.(23) The lower rates of counseling on facility delivery, breastfeeding and postpartum visits in our setting could be because the receipt of financial incentives for the safe motherhood program is contingent on facility delivery and it is assumed these messages will be delivered at that time. However, rates of facility delivery in Nepal are still quite low, 56% in 2016,(20) therefore these aspects of counseling should also be covered during antenatal care. Additionally, a recent review also found major gaps in quality of counseling messaging in Nepal and four other South Asian countries.(31) We did not measure the quality of the counseling messaging themselves, only their delivery. However, Torlesse et al.'s findings may mean that the coverage measures in our population may overestimate the proportion of women who received quality counseling that led to information transfer from provider to client.

For peer review only - http://bmjopen.bmj.com/site/about/guidelines.xhtml

Aside from weight and blood pressure measurement, which should be completed at every visit, there were significant differences in the proportion of women receiving a service at a given gestational age. This corresponds with findings from studies in Pakistan and Bangladesh.(28, 32) The timing is a critical component of care because certain screening tests (i.e., blood test for syphilis) and IFA supplementation should be received earlier in pregnancy.(3) Furthermore, this has implications for women who do not attend ANC during all recommended months and may miss components of care.

The definition of quality of antenatal care is commonly the receipt of certain services at any point during pregnancy. Our data shows that using this definition, 49.1% of the women in our population received good quality of antenatal care. This is higher than another study using 2011 Nepal DHS data,(23) and studies conducted India, Bangladesh and in sub-Saharan Africa.(27, 28, 33, 34) A possible explanation could be that as our data is more recent, health service delivery has improved since the previous studies. Additionally, the other studies primarily rely on maternal recall of services received during ANC, many of which have yet to be validated, which could lead to reporting errors. Finally, our coverage of QOC1 could be higher due to the observer effect (discussed in full in limitations), resulting in the providers at the study clinics providing more complete care than they would if not under observation.

To our knowledge, this is the first study in a low-and-middle-income setting to examine quality of care by including frequency of service provision as outlined by the FANC model. A study in Kenya asked women to report whether they had their weight and blood pressure measured “never, a few times, most of the time and all the time” and found the majority of women to have received the two services most or all of the time.(29) This is comparable to our findings, but this study did not create a final QOC metric to which we can compare our QOC4+ results.

Our analysis showed that the proportion of women received good quality of care differed by health post, more so for QOC4+ than for QOC1. A multi-level model with a random intercept for health post and inclusion of health post level covariates would have been ideal for further elucidating reasons for these differences. However, with only five health posts, the number of clusters was too small to conduct these analyses. Exploring associations between provider characteristics and quality of care would be helpful to explain differences in provision, however the parent study did not collect this information. Distance from facility, which has been shown to be associated with ANC attendance, was also a potential influential factor that we were unable to capture.

The total number of ANC visits is the only characteristic that is significantly associated with both of the QOC definitions, which is consistent with another study.(33) The greater the number of visits a woman attended, the greater the opportunity to have received all necessary components. Younger women (<20 years) were more likely to have received better quality of care, which was not the case in other studies in Nepal and Kenya.(23, 29)

For peer review only

For peer review only - http://bmjopen.bmj.com/site/about/guidelines.xhtml

There were no differences in QOC by SES quartile in our study, whereas others have shown there to be a significant relationship between SES and QOC.(23, 33, 35) However, these studies primarily use data from population-based surveys (e.g., DHS) and have a more heterogeneous population in regard to SES. In our population, the mean score was 6.3 (SD=2.3) and was skewed towards lower scores. There was no difference in QOC1 by education, which has been demonstrated in some studies (29, 36) but not others.(23, 27, 33) As with socioeconomic status, our population was more homogenous in educational attainment; 60% of the population reported no years of education and only one woman reported schooling beyond grade school, compared to 38.9% with no education and 7% with tertiary education in the 2011 Nepal DHS study.(23)

There were no statistically significant associations between covariates and QOC4+, which could be due to a lack of power (discussed further in limitations), as the magnitude of the coefficient for many is quite large. While we cannot draw conclusions on associations between the covariates and the QOC4+ outcome, the descriptive analyses demonstrate the gaps in care and difference in coverage between QOC4+ and QOC1. The health post with the greatest number of patients had no women with high quality QOC4+, driven primarily by a lack of counseling. This health post also had the smallest proportion of women who received 120+ tablets and deworming medication. The increased patient load could explain the low proportions of women receiving the commodity-based services; stock outs might have occurred, or the providers may have limited the number of IFA tablets given to a woman at any one visit to delay a stockout. The lack of counseling could be due to time limitations; the provider may feel that they cannot spend adequate time to convey all the health education messaging because of waiting patients. However, the satisfaction score for this health post was high and similar to the other posts.

A strength of this study is that the receipt of services was collected by direct observation, rather than maternal report. The use of direct observation removes the possibility for recall bias and social desirability bias from the participant, both of which can be present in the cross-sectional studies commonly used in antenatal quality of care studies. Furthermore, the longitudinal design of the parent study allowed for the observation of a woman's entire pregnancy and record of frequency of service provision. Another strength includes the use of trained observers who were required to meet inter and intra-observer reliability standards before the study began.

There are limitations to this analysis as well. While the data is not subject to recall or social desirability bias, the presence of the study observers during ANC could have elicited an observer effect, where the providers knew they were being observed and altered their care. To reduce this possibility, providers were told during the consent process that observers are not medically trained and will not know if the procedures they are observing are correct or not, nor will they report what they observe to the clinic supervisor. Additionally, the observers were present for over a year and one would hope that any observer effect would dissipate over the extended time period. The client satisfaction data was collected at the postpartum interview, which means it is subject to

For peer review only - http://bmjopen.bmj.com/site/about/guidelines.xhtml

recall and social desirability bias. However, there has been evidence of "courtesy bias", where individuals are less likely to report critically at the facility, which is avoided in this case. Additionally, gestational age estimation was based on maternal report of last menstrual period, which is subject to recall bias as well. Eighty-one percent of the sample presented by the fourth month of pregnancy, so the hope is that with a shorter recall period the LMP data is still fairly accurate. Another limitation was that we only observed women who presented for antenatal care at government health facilities. Therefore, the generalizability may be limited to women who attend antenatal care or those who remain within the system in which we observed (excluding private facilities, traditional healers, etc,). In 2016, 16% of women did not receive ANC for a skilled provider, indicating that there is a substantial proportion of the population to which these findings may not be generalizable. (20) A final limitation is the smaller sample size compared other ANC QOC studies, which may have led to limited power in detecting associations between QOC and maternal and facility characteristics. However, because the longitudinal direct observation design is so time and resource intensive, it would be difficult to get a much larger sample size.

This analysis demonstrates that measuring quality of care by receipt of services at least once during pregnancy may be overestimating the true coverage of quality of antenatal care. Furthermore, as countries transition to the eight-visit 2016 WHO ANC recommendations, the degree of underestimation will only increase. Additionally, despite a common belief that commodity-based services such as IFA tablets are often the limiting factor for quality of care, in this study the largest gap in service provision was seen for adequate counseling messaging and the counseling adequacy varied significantly across the study health posts. Future programming and policy efforts should fortify provider training on effective counseling delivery and interpersonal skills to close the gap in counseling provision. This analysis supports the continued research and programmatic efforts in strengthening of routine health information systems and electronic health records to measure quality of care over a woman's entire pregnancy. This would allow the frequency of service provision to be included in quality of care assessment. Additionally, these systems and household surveys should be updated to capture additional indicators of counseling delivery. The inclusion of frequency of specific services provided in ANC in quality of care measurement may be the next step in closing the "quality-coverage" gap and achieving optimal maternal, newborn and child survival.

Conflict of interest: EB, MM, TPL, SK, SLC and JK have no conflicts of interest.

Contribution statement: MM and JK designed the study. TPL, SKK, SL, JK and EB contributed to the implementation of the study. EB conducted analyses and wrote the first draft of manuscript. MM and JK submitted edits to the manuscript. MM, JK, TPL, SKK, EB and SL all have read and approve of the final version of the manuscript.

For peer review only - http://bmjopen.bmj.com/site/about/guidelines.xhtml

Funding: This research was funded by the Bill and Melinda Gates Foundation (grant number OPP1172551). The funders had no role in the design or implementation of the study, nor the analysis or interpretation of the data. JK and MM were the funding recipients

Data sharing: Data is available by emailing Joanne Katz, ScD or Emily Bryce, PhD

Figure legends/caption:

Figure 1 caption: *Source: Tuncalp, Ö et al. Quality of care for pregnant women and newborns-the WHO vision(14)*

Figure 2a and 2b: N/A

Figure 3a legend:

	Deworming		Urine sample
	Blood sample		Weight
	Blood Pressure		2+ counseling
	Any IFA		Discuss problems
	Respectful care		

Figure 3b legend:

	Deworming		Urine sample
	Blood sample		Weight
	Blood Pressure		≥ 4 counseling
	≥ 120 IFA		Discuss problems
	Respectful care		

References

1. Trends in maternal mortality 2000 to 2017: estimates by WHO, UNICEF, UNFPA, World Bank Group and the United Nations Population Division. Geneva: World Health Organization; 2019.

2. Carroli G, Rooney C, Villar J. How effective is antenatal care in preventing maternal mortality and serious morbidity? An overview of the evidence. Paediatric and perinatal epidemiology. 2001;15 Suppl 1:1-42.

3. WHO. WHO recommendations on antenatal care for a positive pregnancy experience. Geneva, Switzerland; 2016.

For peer review only

4. Kuhnt J, Vollmer S. Antenatal care services and its implications for vital and health outcomes of children: evidence from 193 surveys in 69 low-income and middle-income countries. BMJ Open. 2017;7(11):e017122.
5. Neupane S, Nwaru BI. Impact of prenatal care utilization on infant care practices in Nepal: a national representative cross-sectional survey. European journal of pediatrics. 2014;173(1):99-109.
6. Guliani H, Sepehri A, Serieux J. What impact does contact with the prenatal care system have on women's use of facility delivery? Evidence from low-income countries. Soc Sci Med. 2012;74(12):1882-90.
7. Bloom SS, Lippeveld T, Wypij D. Does antenatal care make a difference to safe delivery? A study in urban Uttar Pradesh, India. Health Policy Plan. 1999;14(1):38-48.
8. Villar J, Ba'aqeel H, Piaggio G, et al. WHO antenatal care randomised trial for the evaluation of a new model of routine antenatal care. Lancet (London, England). 2001;357(9268):1551-64.
9. Pandey S. Women's knowledge about the conditional cash incentive program and its association with institutional delivery in Nepal. PloS one. 2018;13(6):e0199230.
10. WHO. WHO antenatal care randomized trial: manual for the implementation of the new model. Geneva; 2002.
11. Jahn A, Dar Iang M, Shah U, et al. Maternity care in rural Nepal: a health service analysis. Tropical medicine & international health : TM & IH. 2000;5(9):657-65.
12. Hodgins S, D'Agostino A. The quality-coverage gap in antenatal care: toward better measurement of effective coverage. Glob Health Sci Pract. 2014;2(2):173-81.
13. Bhutta ZA, Darmstadt GL, Hasan BS, et al. Community-based interventions for improving perinatal and neonatal health outcomes in developing countries: a review of the evidence. Pediatrics. 2005;115(2 Suppl):519-617.
14. Tuncalp, Were WM, MacLennan C, et al. Quality of care for pregnant women and newborns-the WHO vision. BJOG : an international journal of obstetrics and gynaecology. 2015;122(8):1045-9.
15. Raven JH, Tolhurst RJ, Tang S, et al. What is quality in maternal and neonatal health care? Midwifery. 2012;28(5):e676-83.
16. Donabedian A. Exploratings in quality assessment and monitoring definition of quality and approaches to its assessment. 1980.
17. Donabedian A. The quality of care. How can it be assessed? Jama. 1988;260(12):1743-8.
18. Hulton L, Matthews Z, Stones RW. A framework for the evaluation of quality of care in maternity services. 2000.
19. Bruce J. Fundamental elements of the quality of care: a simple framework. Stud Fam Plann. 1990;21(2):61-91.
20. Nepal MoH. Nepal Demographic and Health Survey 2016. Kathmandu, Nepal: Ministry of Health, Nepal; 2017.
21. Aryal K, Sharad SK, Mukti NK, Bihungum B, Shiv SL, Shambhu K, and Mona SM. Maternal Health Care in Nepal: Trends and Determinants. Rockville, Maryland, USA: ICF; 2019.
22. Lama TP, M. Chaulagain, A. Rai, and S. Gurung. Assessment of the Quality of Antenatal Care, Family Planning, and Sick Child Care Services in Nepal: Further Analysis of the 2015 Nepal Health Facility Survey. Rockville, Maryland, USA: ICF; 2019.

For peer review only - http://bmjopen.bmj.com/site/about/guidelines.xhtml

23. Joshi C, Torvaldsen S, Hodgson R, et al. Factors associated with the use and quality of antenatal care in Nepal: a population-based study using the demographic and health survey data. BMC pregnancy and childbirth. 2014;14:94.
24. Population MoHa. Annual Report, Department of Health Services 2074/2075 (2017/2018). Kathmandu, Nepal; 2019.
25. Population MoHa. Annual Report, Department of Health Services 2070/71 (2013/2014). Kathmandu, Nepal; 2015.
26. Sheffel A, Zeger S, Heidkamp R, et al. Development of summary indices of antenatal care service quality in Haiti, Malawi and Tanzania. BMJ Open. 2019;9(12):e032558.
27. Islam MM, Masud MS. Determinants of frequency and contents of antenatal care visits in Bangladesh: Assessing the extent of compliance with the WHO recommendations. PloS one. 2018;13(9):e0204752.
28. Siddique AB, Perkins J, Mazumder T, et al. Antenatal care in rural Bangladesh: Gaps in adequate coverage and content. PloS one. 2018;13(11):e0205149.
29. Afulani PA, Buback L, Essandoh F, et al. Quality of antenatal care and associated factors in a rural county in Kenya: an assessment of service provision and experience dimensions. BMC Health Serv Res. 2019;19(1):684.
30. Marchant T, Tilley-Gyado RD, Tessema T, et al. Adding content to contacts: measurement of high quality contacts for maternal and newborn health in Ethiopia, north east Nigeria, and Uttar Pradesh, India. PloS one. 2015;10(5):e0126840.
31. Andrews RM, McCarthy J, Carapetis JR, et al. Skin Disorders, Including Pyoderma, Scabies, and Tinea Infections. Pediatric Clinics of North America. 2009;56(6):1421-40.
32. Agha S, Tappis H. The timing of antenatal care initiation and the content of care in Sindh, Pakistan. BMC pregnancy and childbirth. 2016;16(1):190.
33. Olorunsaiye CZ, Brunner Huber LR, Laditka SB, et al. Individual and community socioeconomic factors related to the quality of antenatal care: a multilevel analysis of West and Central Africa. Women Health. 2021;61(1):15-26.
34. Singh L, Dubey R, Singh S, et al. Measuring quality of antenatal care: a secondary analysis of national survey data from India. BJOG : an international journal of obstetrics and gynaecology. 2019;126 Suppl 4:7-13.
35. Sharma J, Leslie HH, Kundu F, et al. Poor Quality for Poor Women? Inequities in the Quality of Antenatal and Delivery Care in Kenya. PloS one. 2017;12(1):e0171236.
36. Lee E, Madhavan S, Bauhoff S. Levels and variations in the quality of facility-based antenatal care in Kenya: evidence from the 2010 service provision assessment. Health Policy Plan. 2016;31(6):777-84.

Table 1

	FANC	Nepal MoH annual report	Nepal DHS analyses	Expert survey	What CVS-2 captured

For peer review only - http://bmjopen.bmj.com/site/about/guidelines.xhtml

Provision of care					
IFA	X	X	X	X	X
IPTp	X			(not for Nepal)	
TT vaccine	X	X	X	X	
Deworming		X	X	X	X
BP measured	X	X (2017 report)	X	X	X
Weight measured			X		X
Blood sample	X		X	X	X
Urine sample	X		X	X	X
Counseling	X	X	X	X	X
Nutrition	X	X	X	X	X
Breastfeeding	X			X	X
Facility delivery	X	X	X		X
Family planning			X		
PP care		X			X
Experience of care					
Ability to discuss problems			X	X	X
Respectful care			X	X	X
Satisfied with explanation of information			X	X	

Table 2. Quality of care definitions

	Definition 1: Entire study population (QOC1)	Definition 2: Women who had recommended 4+ visits (QOC4+)
Provision of care		
IFA	Any	120+ (30/visit)**
Deworming	Once	Once
BP measured	Once	Four times**
Weight measured	Once	Four times**
Blood sample prescription	Once	Once
Urine sample prescription	Once	Once
Counseling*	Two of four areas	All four areas
Experience of care		

For peer review only

For peer review only - http://bmjopen.bmj.com/site/about/guidelines.xhtml

Ability to discuss problems	"Yes"	"Yes"
Respectful care	"Yes"	"Yes"

*Areas of counseling: nutrition, breastfeeding, facility delivery and PP care
**IFA provision, weight and BP measurement are included at each visit in the FANC model. Other references do not indicate frequency of provision throughout pregnancy

Table 3. Characteristics of participants

Characteristic	Mean	SD	Range
Woman's age, years	22.5	4.2	16 to 41
Estimated GA at enrollment, months	3.5	1.3	1 to 9
Total number of visits per woman	4.5	2.5	1 to 14
	N	%	
SES quartiles*			
1	167	38%	
2	74	17%	
3	132	30%	
4	61	14%	
Did this woman have any prior live births?			
Yes	298	69%	
No	136	31%	
Did the woman have any years of education?			
No	259	60%	
Yes	175	40%	
Trimester at enrollment			
1-3 months	190	44%	
4-6 months	236	55%	
7-9 months	7	2%	
Total number of visits per woman			
1 visit	46	11%	
2-4 visits	205	47%	
5-7 visits	125	29%	
8+ visits	58	13%	
Number of visits during recommended months (<4, 4-6, 7-8, 9+)			
1	74	17%	
2	100	23%	
3	126	29%	
4	133	31%	

Table 4. Distribution of provision & experience of care during ANC

For peer review only - http://bmjopen.bmj.com/site/about/guidelines.xhtml

Provision of care	Never	Once	More than once
IFA or medicine-related			
Received IFA tablets	8.3%	19.8%	71.9%
Received a deworming	25.6%	73.5%	0.9%
Was given or told to buy calcium	13.8%	26.3%	59.9%
Physical exam			
Blood pressure was measured	2.8%	19.1%	78.1%
Weight measured	1.6%	17.1%	81.3%
Was given a prescription for a blood sample	6.7%	59.0%	34.3%
Was given a prescription for a urine sample	7.6%	55.5%	36.9%
Counseling			
Was told why deworming was being given	93.8%	6.2%	0.0%
Was told anything about blood pressure measurement	4.4%	21.0%	74.7%
Was told anything about weight gain or loss	2.1%	19.1%	78.8%
Was told why biospecimen (urine or blood) should be given	8.5%	42.9%	48.6%
Was given advice about food or nutrition during pregnancy	5.3%	19.4%	75.3%
Was told not drink and/or smoke	49.8%	27.9%	22.4%
Was given advice about breastfeeding	70.5%	7.1%	22.4%
Was told to deliver in a facility	28.3%	32.3%	39.4%
Was given advice on postpartum-related subjects	67.5%	11.8%	20.7%
Experience of care	N (%)		
Reported being able to discuss problems and concerns	379 (87.3%)		
Reported level of respect of treatment by health provider			
Very disrespectful	2 (0.5%)		
Somewhat disrespectful	2 (0.5%)		
Neither disrespectful nor respectful	5 (1.2%)		
Somewhat respectful	135 (31.0%)		
Very respectful	290 (66.8%)		

Table 5. Receipt by gestational age at visit

Service observed	Less than 4 months	4-6 months	7-8 months	9+ months	Chi-square

For peer review only - http://bmjopen.bmj.com/site/about/guidelines.xhtml

					p-value
IFA or medicine related					
Received IFA tablets	20.90%	66.40%	69.10%	69.00%	p <0.01
Woman was given deworming tablets	20.60%	30.50%	0.90%	0.00%	p <0.01
Woman was given or told to buy calcium tablets	19.70%	51.10%	53.20%	45.60%	p <0.01
Physical exam					
Woman's blood pressure was measured	69.60%	65.80%	67.50%	70.80%	0.335
Woman's weight was measured	69.00%	68.40%	68.20%	69.90%	0.958
Woman was given a prescription for a blood sample	56.40%	36.50%	16.60%	13.40%	p <0.01
Woman was given a prescription for a urine sample	56.10%	37.20%	17.30%	13.40%	p <0.01
Counseling					
Woman was told why deworming tablet was given	3.00%	1.60%	0.50%	0.60%	0.015
Woman was told anything about her BP	66.00%	61.20%	62.70%	63.80%	0.479
Woman was told anything about weight/gain/loss	67.50%	65.80%	64.10%	65.70%	0.805
Was told why biospecimen (urine or blood) should be given	53.40%	41.70%	25.80%	24.30%	p <0.01
Woman was given advice about food/nutrition during pregnancy	72.50%	64.00%	61.30%	60.20%	p <0.01
Received counseling on smoking and/or drinking	29.60%	22.30%	13.10%	16.70%	p <0.01
Woman was given any advice on breastfeeding	14.30%	12.10%	16.40%	28.90%	p <0.01
Woman was told to deliver in a facility	28.40%	25.60%	27.20%	61.10%	p <0.01
Received counseling on postpartum-related subjects	14.90%	13.00%	15.90%	28.60%	p <0.01

For peer review only - http://bmjopen.bmj.com/site/about/guidelines.xhtml

Table 6. Regression results

	Definition 1 (QOC1): Total study population (N=434)		Definition 2 (QOC4+): Among women with 4+ visits N=(262)	
	Bivariate model	Multivariate model	Bivariate model	Multivariate model
	RR (95% CI)	aRR (95% CI)	RR (95% CI)	aRR (95% CI)
Maternal age ≤ 20	1.14 (0.94-1.38)	1.27* (1.03-1.58)	0.98 (0.47-2.02)	1.53 (0.69-3.43)
No prior live birth	1.00 (0.82-1.24)	0.81 (0.64-1.02)	0.70 (0.31-1.60)	0.60 (0.23-1.56)
Any years of education	1.10 (0.91-1.34)	0.96 (0.80-1.16)	1.05 (0.51-2.16)	0.62 (0.31-1.26)
Number of visits	1.07** (1.04-1.11)	1.18** (1.13-1.23)	0.96 (0.80-1.15)	1.46** (1.11-2.80)
Enrollment during first trimester	1.03 (0.85-1.25)	1.22* (1.01-1.49)	1.07 (0.52-2.18)	1.15 (0.63-2.08)
SES quartiles (ref: 1st)				
2	1.27 (0.97-1.67)	1.16 (0.92-1.47)	0.67 (0.19-2.42)	1.77 (0.52-6.03)
3	1.21 (0.95-1.54)	0.99 (0.79-1.24)	1.77 (0.76-4.10)	1.48 (0.64-3.40)
4	1.31 (0.99-1.74)	1.09 (0.82-1.45)	1.22 (0.39-3.82)	1.17 (0.37-3.71)
Health post (ref: 1)				
2	0.80 (0.56-1.12)	0.66* (0.48-0.91)	---[1]	---[1]
3	1.62** (1.18-2.23)	1.69** (1.23-2.30)	24.73** (3.46-176.86)	36.11** (5.03-259.10)
4	1.63** (1.22-2.19)	1.84** (1.40-2.41)	7.68* (1.00-59.11)	12.59* (1.67-94.82)
5	1.55* (1.10-2.18)	1.95** (1.39-2.73)	2.82 (0.19-42.70)	6.22 (0.47-81.92)

*- $p<0.05$
**- $p<0.01$
[1]- There were no women who received good quality of care by the QOC4+ definition

For peer review only

Figure 1

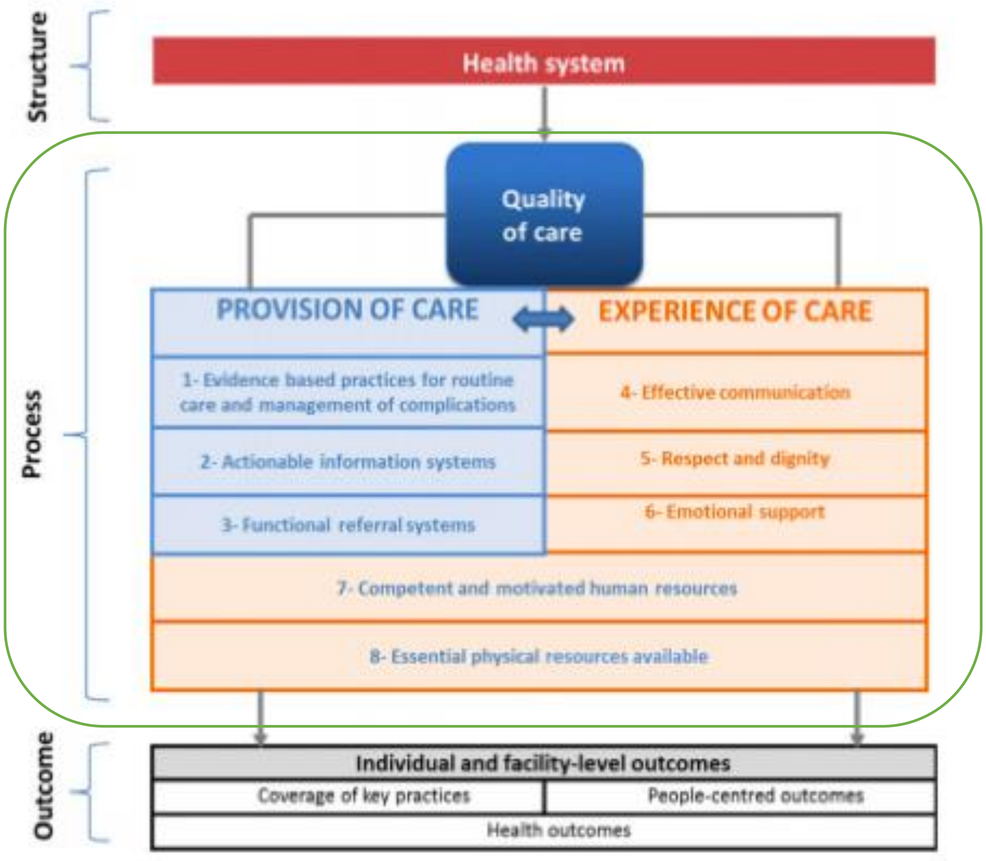
Structure
Health system
Process
Quality of care
PROVISION OF CARE
EXPERIENCE OF CARE
1- Evidence based practices for routine care and management of complications
2- Actionable information systems
3- Functional referral systems
4- Effective communication
5- Respect and dignity
6- Emotional support
7- Competent and motivated human resources
8- Essential physical resources available
Outcome
Individual and facility-level outcomes
Coverage of key practices
People-centred outcomes
Health outcomes

Figures 2a. QOC1 and 2b. QOC4+

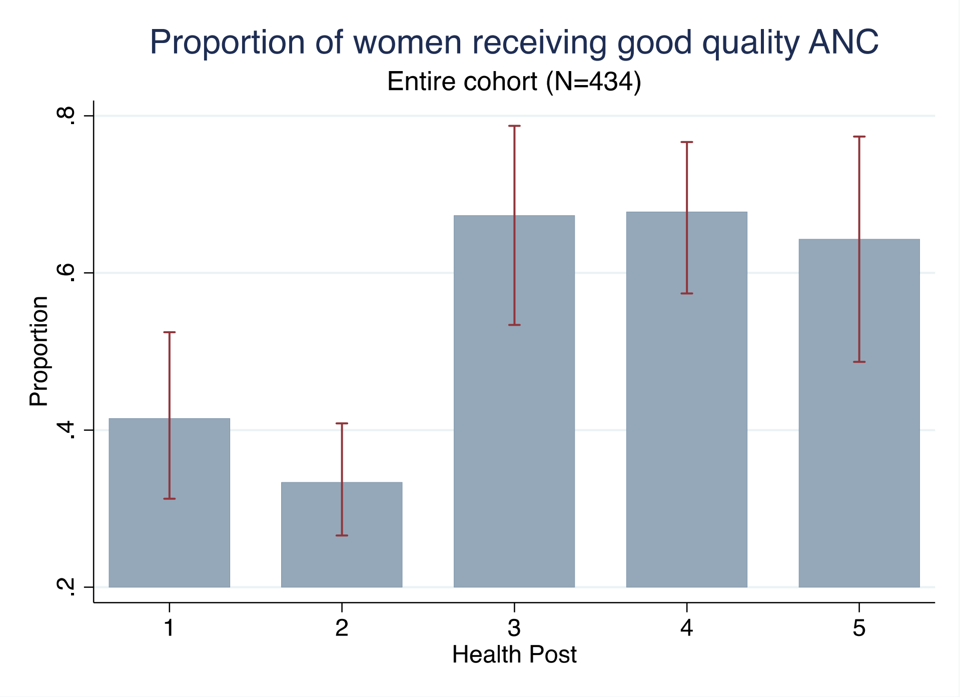

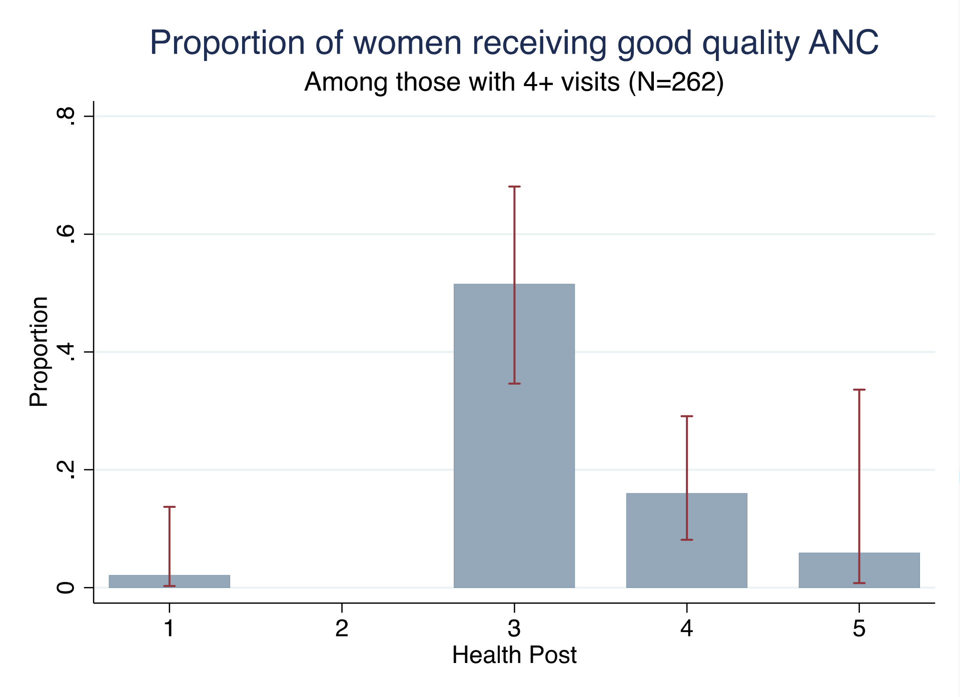

Figures 3a. QOC1

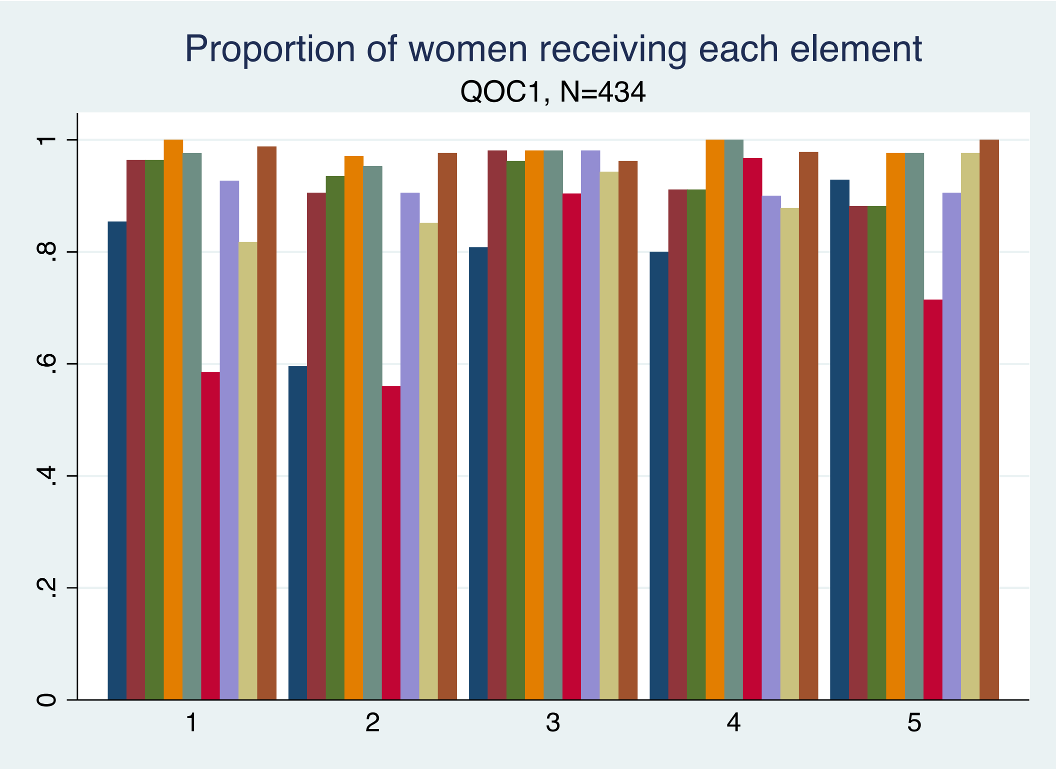

Figure 3b. QOC4+

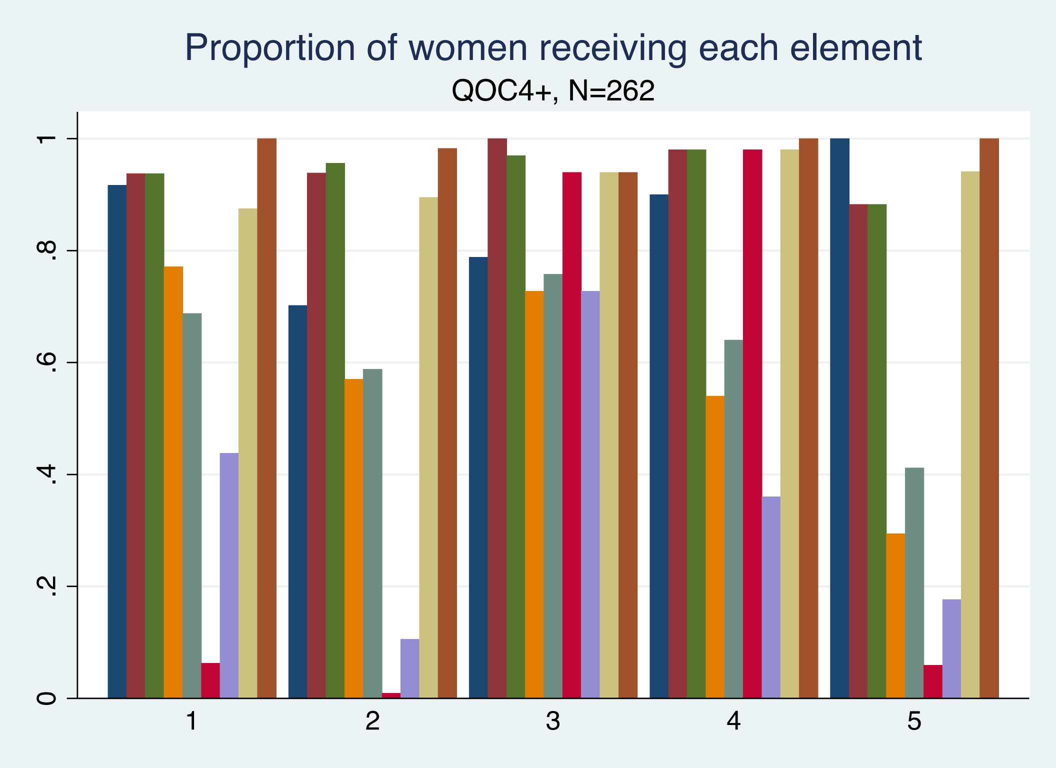

For peer review only

STROBE Statement—Checklist of items that should be included in reports of ***cohort studies***

	Item No	Recommendation	Page No
**Title and abstract**	1	(*a*) Indicate the study's design with a commonly used term in the title or the abstract<br>(*b*) Provide in the abstract an informative and balanced summary of what was done and what was found	1, 2
**Introduction**			
Background/rationale	2	Explain the scientific background and rationale for the investigation being reported	3-5
Objectives	3	State specific objectives, including any prespecified hypotheses	5
**Methods**			
Study design	4	Present key elements of study design early in the paper	5
Setting	5	Describe the setting, locations, and relevant dates, including periods of recruitment, exposure, follow-up, and data collection	5
Participants	6	(*a*) Give the eligibility criteria, and the sources and methods of selection of participants. Describe methods of follow-up<br>(*b*) For matched studies, give matching criteria and number of exposed and unexposed	5
Variables	7	Clearly define all outcomes, exposures, predictors, potential confounders, and effect modifiers. Give diagnostic criteria, if applicable	6
Data sources/ measurement	8*	For each variable of interest, give sources of data and details of methods of assessment (measurement). Describe comparability of assessment methods if there is more than one group	5-7
Bias	9	Describe any efforts to address potential sources of bias	11
Study size	10	Explain how the study size was arrived at	5
Quantitative variables	11	Explain how quantitative variables were handled in the analyses. If applicable, describe which groupings were chosen and why	6-7
Statistical methods	12	(*a*) Describe all statistical methods, including those used to control for confounding<br>(*b*) Describe any methods used to examine subgroups and interactions<br>(*c*) Explain how missing data were addressed<br>(*d*) If applicable, explain how loss to follow-up was addressed<br>(*e*) Describe any sensitivity analyses	7
**Results**			
Participants	13*	(a) Report numbers of individuals at each stage of study—eg numbers potentially eligible, examined for eligibility, confirmed eligible, included in the study, completing follow-up, and analysed<br>(b) Give reasons for non-participation at each stage<br>(c) Consider use of a flow diagram	7
Descriptive data	14*	(a) Give characteristics of study participants (eg demographic, clinical, social) and information on exposures and potential confounders<br>(b) Indicate number of participants with missing data for each variable of interest<br>(c) Summarise follow-up time (eg, average and total amount)	7-8
Outcome data	15*	Report numbers of outcome events or summary measures over time	8

For peer review only - http://bmjopen.bmj.com/site/about/guidelines.xhtml

For peer review only

Main results	16	(*a*) Give unadjusted estimates and, if applicable, confounder-adjusted estimates and their precision (eg, 95% confidence interval). Make clear which confounders were adjusted for and why they were included<br>(*b*) Report category boundaries when continuous variables were categorized<br>(*c*) If relevant, consider translating estimates of relative risk into absolute risk for a meaningful time period	8-9
Other analyses	17	Report other analyses done—eg analyses of subgroups and interactions, and sensitivity analyses	NA
**Discussion**			
Key results	18	Summarise key results with reference to study objectives	9
Limitations	19	Discuss limitations of the study, taking into account sources of potential bias or imprecision. Discuss both direction and magnitude of any potential bias	11
Interpretation	20	Give a cautious overall interpretation of results considering objectives, limitations, multiplicity of analyses, results from similar studies, and other relevant evidence	9-11
Generalisability	21	Discuss the generalisability (external validity) of the study results	12
**Other information**			
Funding	22	Give the source of funding and the role of the funders for the present study and, if applicable, for the original study on which the present article is based	1

*Give information separately for exposed and unexposed groups.

Note: An Explanation and Elaboration article discusses each checklist item and gives methodological background and published examples of transparent reporting. The STROBE checklist is best used in conjunction with this article (freely available on the Web sites of PLoS Medicine at http://www.plosmedicine.org/, Annals of Internal Medicine at http://www.annals.org/, and Epidemiology at http://www.epidem.com/). Information on the STROBE Initiative is available at http://www.strobe-statement.org.

For peer review only
